# The effect of Wnt/β-catenin signaling on PD-1/PDL-1 axis in HPV-related cervical cancer

**DOI:** 10.32604/or.2022.026776

**Published:** 2023-01-12

**Authors:** PARISA SHIRI AGHBASH, NIMA HEMMAT, BEHZAD BARADARAN, AHAD MOKHTARZADEH, VAHDAT POORTAHMASEBI, MAHIN AHANGAR OSKUEE, HOSSEIN BANNAZADEH BAGHI

**Affiliations:** 1 Immunology Research Center, Tabriz University of Medical Sciences, Tabriz, 5165665931, Iran; 2 Infectious and Tropical Diseases Research Center, Tabriz University of Medical Sciences, Tabriz, 5165665931, Iran; 3 Department of Immunology, Faculty of Medicine, Tabriz University of Medical Sciences, Tabriz, 5165665931, Iran; 4 Department of Virology, Faculty of Medicine, Tabriz University of Medical Sciences, Tabriz, 5165665931, Iran

**Keywords:** Cervical cancers, HPV-related cancers, PD-1, PD-L1, PD-L2, Wnt/β-catenin pathway

## Abstract

Infection with high-risk human papillomavirus (HPV), including HPV-16 and HPV-18, is the main cause of malignancies, such as cervical cancer. Viral oncoproteins encoded by HPV are expressed in HPV-positive cancers and associated with the early cancer stages and the transformation of normal cells. The signaling pathways involved in the transformation of normal cells to cancerous form and the subsequently expressed programmed cell death-ligand 1 (PD-L1) on the surface of the transformed cells lead to a disruption in recognition of tumor cells by the immune cell system, including T lymphocytes and dendritic cells which lead to the development of cervical cancer malignancy. These cells also produce modest levels of cytokines during exhaustion, tumor-infiltrating T CD4+ cells with high levels of PD-1 and CD39 release considerable quantities of cytokines. The Wnt/β-catenin signaling pathway, which controls the expression of genes involved in the tumor cells’ markers, is demonstrated to be one of the most potent cancer stimulants. It leads to the evasion of the tumor cells from immune cell detection and ultimately avoids being recognized by dendritic cells or T-cells. PD-L1, as an inhibitory immune checkpoint, is essential for controlling immune system activity by inhibiting T-cells’ inflammatory function. In the present review, we looked into how Wnt/β-catenin affects the expression of PD-L1 and related genes like c-MYC in cancer cells and its role in the development of HPV-induced malignancy. We hypothesized that blocking these pathways could be a potential immunotherapy and cancer prevention method.

## Introduction

Cells determine their function through signaling pathways and communication with other cells and their microenvironment. Due to ligands’ binding to their receptors on the cell surface, protein cascades are activated and consequently affect the level of gene transcription, leading to the conversion of external stimuli into biochemical signals. These signaling pathways control biological effects such as proliferation, differentiation, and death; thus, disrupting these pathways leads to malignancy [1]. Also, they are owing to changes in some critical cell proliferation and apoptosis-controlling pathways, including phosphatidylinositol 3-kinase (PI3K)/phosphorylation of protein kinase B (AKT), extracellular signal-regulated kinase (ERK)/mitogen-activated protein kinase (MAPK), Notch, Wnt/β-catenin, and epidermal growth factor receptor (EGFR). In addition, a variety of inhibitory immune checkpoint molecules, such as programmed cell death protein 1 (PD-1, CD279) and its ligand PD-L1 (CD274), have been playing a significant role in various types of cancers and chronic viral infections [1–3]. For instance, it was demonstrated in a report published on the PD-1/PD-L1 axis in acute viral infections affecting the lower respiratory tract that this pathway impairs the activity of CD8+ T-cells in the human respiratory system. In addition, dendritic cells inhibit T-cell function in acute viral infections by highly expressing PD-L1 on their surfaces [3]. PD-1, on the other hand, is expressed in lymphocytes, specifically CD8+ and CD4+ T-cells, and is an inducible negative regulator of T-cell activity; as a result, PD-L1 is one of two PD-1 ligands found on antigen-presenting cells (APCs), peripheral tissues, and cancerous cells [4]. As a result, the interaction of PD-1/PD-L1 leads to the activation of inhibitory signals in T-cells, which eventually leads to the destruction of T-cells, anergy, and reduced function [5,6]. PD-1 causes regulatory T-cells to face less apoptosis while increasing the death of some T-cells in lymph nodes. On the surface of CD8+ and CD4+ T-cells, PD-1 is more apparent. The PD-1/PDL1 pathway reduces the activity of T-cells; this inhibitory effect is done by binding PD-1 to its ligands (PD-L1 and PD-L2), which are produced on the surface of cancerous cells and peripheral tissues. The expression of PD-L1 also regulates IFN-γ-secreting T-cells [3]. Moreover, it has been illustrated that the interaction of PD-1/PD-L1 can increase the transcription of several genes involved in cancer induction. Cancer cells express immune system regulators such as the cluster of differentiation (CD)-47 and PD-L1 to induce immune response resistance. Also, cellular myelocytomatosis (c-MYC) activation is due to CD-47 and PD-L1 expression [4]. Because c-MYC can directly bind to the CD-47 and PD-L1 gene promoters, inhibiting c-MYC reduces the expression of both genes (CD-47 and PD-L1) and thus improves the anti-tumor immune response [7]. It is hypothesized that T-cell apoptosis is associated with c-MYC overexpression, a critical target gene of the Wnt/β-catenin signaling pathway [8]. Additionally, Wnt/β-catenin signaling has been implicated as one of the primary inducers of cancer, accelerating the development of the disease through controlling the tumor immune cycle in various cell types, including dendritic cells, T-cells, and tumor cells [4]. Some significant regulators of the anti-tumor function of T-cells, especially effective T-cells, T helper cells (Th), and regulatory T-cells (T-reg), are changed functionally by abnormal Wnt/β-catenin signaling [4]. Besides, the activation of the Wnt/β-catenin signaling pathway is observed during various types of malignancies, such as hepatocellular carcinoma, human immunodeficiency virus (HIV), breast cancer, as well as in HPV-related cancers such as oropharyngeal and cervical cancers, head and neck, anal cancer, and vulvar cancer [1]. As mentioned above, increased levels of PD-1 expression on the T-cells and APC cells, and also hyper-activation of the Wnt pathway during chronic HPV infection, are positively associated with tumor cell metastasis and HPV-associated cancers such as cervical cancer and cervical intraepithelial neoplasia (CIN) grade [1,9]. Besides, inhibition of the Wnt signaling pathway in cancer cells could disrupt the expression of CD-47 and PD-L1, resulting in an increased immune defense against tumors [8]. In other words, MYC signaling leads to increased expression of CD47 and PD-L1 in tumor cells. Meanwhile, due to CD47-SIRPα interaction, the phagocytosis activity of tumor cells by macrophages and dendritic cells is disturbed. As a result, the lack of activity of the innate immune system may cause an increase in the recruitment of T-cells to the tumor microenvironment. In contrast to PD-L1 expression, it may disrupt in the function of T-cells in the tumor microenvironment. In addition, the Wnt/β-catenin signaling pathway may affect MYC activation [10].

**Figure 1:**
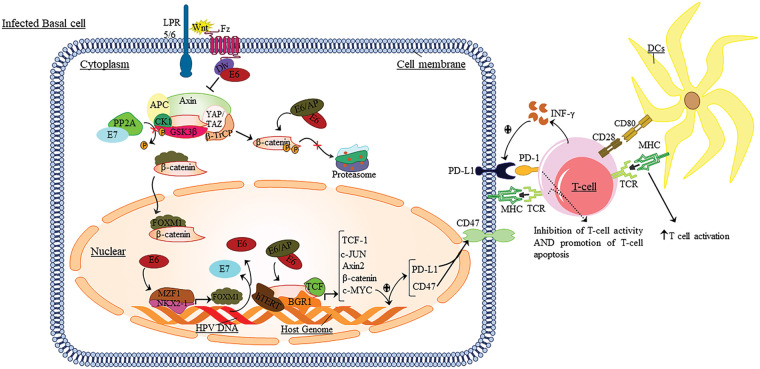
The role of Wnt/β-catenin and PD1/PD-L1 signaling in HPV infection.

The present study hypothesized that current research concentrates on the Wnt/β-catenin and PD-1 signaling pathways, crucial elements in cancers, particularly cancers related to viruses. These findings may alter the manner that individuals evaluate cancer patients and immunotherapy (Fig. 1).

During infection, the PD-1/PD-L1 signaling axis and Wnt play an essential role in developing and maligning epithelial cells. Thus, after presenting the antigen to the T-cells, the DC cells induce signaling pathways in T-cells, resulting in the induction of TCR and the secretion of pro-inflammatory cytokines. Then these cytokines lead to the expression of PD-1 on the surface of T-cells. In this line, Wnt binds to its receptors. The integrated HPV genome regulates these pathways to increase escape and inhibit the immune response against tumor cells by expressing E6 and E7 oncoproteins. Also, these oncoproteins lead to the induction of a series of cellular signaling pathways of Wnt; it eventually conducts the expression of the c-MYC in the target cell nucleus. The c-MYC gene leads to increased expression of PD-L1 and CD47 at the surface of tumor cells. By binding PD-L1 to PD-1 expressed at the surface of T-cells, it leads to apoptosis, anergy, and ultimately inhibition of the immune response *vs*. tumor cells. Also, INF-γ secreted by T-cells leads to increased PD-L1 expression at the surface of tumor cells.

## The Function of Wnt/β-Catenin Signaling Pathway in Cancer

In 1991, the term Wnt was coined from the acronyms homologous wingless (WG) and INT1, which refers to a group of genes that encode secretory glycoproteins [11]. 11 receptors of the Frizzled (Fz) family in humans could activate the Wnt signaling pathway, such as Fz1 to Fz10, Smo, and two low-density lipoproteins receptor-related protein (LRP) 5 and 6 [12]. Wnt ligands activate three active signal transduction pathways, including the canonical Wnt/β-catenin pathway and two non-canonical pathways [13]. According to studies, the Wnt/β-catenin signaling pathway is crucial for cell growth, proliferation, differentiation, adhesion, and polarity [11,14–16], as well as for carcinogenesis from the initial stages to the advanced parts in a variety of neoplasms [17].

The β-catenin accumulation due to the Wnt pathway’s abnormal function is observed in the cytoplasm [18]. Indeed, the binding of Wnt to Fz and low-density lipoprotein receptor-related protein 5/6 (LPR5/6) receptors results in the accumulation of β-catenin in the nucleus and cytoplasm; after Fz dimerization by LRP5/6 co-receptor, the intracellular motif of Fz leads to the absorption of cytoplasmic protein Dishevelled (Dlv) [19]. Then, the LPR5/6, phosphorylated by CK1 and AXIN, binds to the β-catenin destruction complex that eventually prevents the β-catenin destruction that these mechanisms use to promote nuclear β-catenin transfer [18,20–22].

The β-catenin then enters the nucleus as a transcription co-activator, resulting in activated transcription via the complex of transcription factors such as T-cell factor (TCF)/lymphoid enhancing factor (LEF) [17]. In the nucleus, β-catenin binding to TCF/LEF conducts the separation of co-receptors, including Groucho/TLE; as a result, it interacts with co-activators such as cell cycle-related and expression elevated protein in tumor (CREPT), four-and-a-half-LIM-domain 2 (FHL2), and CREB-binding protein (CBP/p300) and remodelers of chromatin such as brahma-related gene 1 (Brg-1) [21,23–29].

Some β-catenin in the cytoplasm are affected by proteasome degradation. During this process, the AXIN scaffold protein controls the process of β-catenin degradation by utilizing important proteins such as glycogen synthase kinase-3β (GSK-3β), casein kinase 1 (CK1), adenomatous polyposis coli (APC), Yes-associated protein (YAP)/Transcriptional co-activator with PDZ-binding motif (TAZ), and beta-transducing repeat-containing protein (β-TrCP) [30–33]. APC inhibits PP2A phosphatase-mediated β-catenin dephosphorylation [34]. It has been demonstrated that the recurrence of E3 β-TrCP ubiquitin ligase by the YAP/TAZ complex and the detection of serine (Ser) or threonine (Thr) phosphorylation by E3 β-TrCP ubiquitin ligase cause the increase in ubiquitination of β-catenin and its subsequent proteasomal destruction [33].

Furthermore, other factors that participate in tumor-genesis, including c-MYC, metalloproteinase 7 (MMP-7), and the vascular endothelial growth factor (VEGF), are stimulated by the β-catenin/TCF-LEF complex [35]. Following the above interactions with catenin, various genes such as c-JUN and Cyclin D1 are expressed, which leads to the regulation of polarity, proliferation, and differentiation of various cellular genes in the development of malignancy [29].

## The Function of PD-1/PD-L1 Signaling Pathway in Cancer

PD-1, as an inhibitory co-stimulant receptor and a member of the CD28 family, is expressed in innate and acquired immune cells and regulates inflammation and self-reactivity [36]. Also, as an inhibitory receptor in T-cells, PD-1 has been associated with T-cells malfunctioning during infectious diseases and malignancies [37]. This inhibitory molecule contains two types of ligands from the B7 family on the cell surface, including PD-L1 (B7-H1) and PD-L2 (B7-DC), which have different expression patterns [38–41]. It is a kind of type 1 transmembrane receptor of the immunoglobulin super family [38]. In many tissues, PD-L1 is expressed on the surface of immune cells such as T-cells, B cells, dendritic cells (DC), macrophages (MQ), and non-hematopoietic parenchymal cells. PD-L2, however, is only expressed on the antigen-presenting cells’ surface, including DCs, MQ, B cells, and many tumors [42–46]. It has been speculated that the regulation of primary T cell function in lymphatic organs and the control of inflammation and autoimmunity in peripheral tissues is achieved by binding B7 family ligands to CD28 family receptors [37]. Moreover, gamma-chain cytokines such as IL-2, IL-7, and IL-15 promote the expression of PD-1 on the surface of monocytes, macrophages, and T-cells [37]. Granulocyte-macrophage colony-stimulating factor (GM-CSF), interferon-gamma (INF-γ), and IL-4 also regulate the expression of PD-L1 and PD-L2 on the surface of macrophages; among these; IL-4 and GM-CSF, have a significant role in the expression of PD-L2 [47,48]. Meanwhile, interferon regulatory factor-1 (IRF-1) binding to the PD-L1 gene promoter is regulated by downstream IFN-γ signaling [49]. It has been demonstrated that mechanisms such as INF-γ production by lymphocytes in the tumor environment, genomic alterations, and the activation of carcinogenic signaling pathways increase PD-L1 expression in tumor cells [50,51].

Furthermore, in a study conducted by Dong et al. [52], in 2018, it was reported that overexpression of miR-18a in cervical cancer indicates an increase in PD-L1 levels by preventing phosphatase and tensin homolog (PTEN) (a PI3K-AKT pathway inhibitor), WNK2 (a MAPK inhibitor), and SRY-box transcription factor 6 (SOX6) (a type of Wnt/β-catenin inhibitor), activated PI3K/AKT, MAPK, and Wnt/β-catenin pathways and promoted the induction of PD-L1 [52]. Moreover, the evidence suggests that over-stimulation of the EGFR pathway seen in EGFR-induced cancers can lead to the expression of PD-L1 in human cancer cells [37,53]. The IFN-γ and EGFR have used Janus kinase 2 (JAK2) as standard signaling to transmit external or internal signals to tumor cells, respectively [54]. For example, in HPV infection individuals, PD-L1 expression is induced by EGFR and JAK2/signal transducer and activator of transcription 1 (STAT1)-dependent procedures; in particular, inhibition of JAK2 prevents re-regulation of PD-L1 in tumor cells, leading the increased immunologicity [54].

It has been denoted that in tumor cells, two main pathways regulate the expression of PD-L1. The first one is an “external” signaling pathway in which an anti-tumor cellular immune response drives INF-γ production via natural killer (NK) cells and CD8+ tumor-infiltrating lymphocytes (TIL). The produced INF-γ contributed to the expression of PD-L1 in tumor cells [54]. The second one is an “intrinsic” signaling pathway in which tumor-induced pathways within the tumor cells induce the over-expression of PD-L1 [55]. Given that the INF-γ produced by NK cells and T-cells is a well-known stimulus for PD-L1 expression, the complex signaling pathways of PD-L1 regulation must be adequately investigated. Regarding the PD-1 ligands that play a significant role in maintaining immune suppressive tumors, a set of PD-1 and PD-L1 expression and signaling control mechanisms are emerging [37,56]. Besides, it demonstrated that this molecule played a significant role in inducing and maintaining central and peripheral tolerance [57]. By presenting the viral antigen via DCs, a signaling pathway is induced through PD-1 on CD8+ T-cells; as a result, it leads to T-cells tolerance. In other words, PD-1 is called a “rheostat,” the T cell response’s threshold, power, and duration [58–60].

## The Impact of the Wnt Signaling Pathway and PD-1/PD-L1 Axis on T CD4+ and T CD8+ Function

The HPV-specific immune response, as well as the destruction of HPV-infected cells in the cervical region, is initiated by T helper cells (CD4+), cytotoxic T-cells (CD8+), and dendritic cells (DC) [61]. As mentioned in the previous section, PD-L1 molecules are overexpressed on the surface of T-cells during viral infection, resulting in the induction of tolerance, inhibition of activity, and cytokine production [5,6].

On the other hand, the Wnt/β-catenin signaling pathway is critical for T-cells proliferation and differentiation; hence, activation of the TCF and LEF genes and the Wnt/β-catenin pathway in mature T-cells causes cytotoxic T cell maturation to be impaired [62]. In addition, the Wnt pathway activates Fra1, a target of the interleukin-17 (IL17RA) cytokine cascade [63,64]. IL-17A, produced by T helper 17 (Th17) cells in response to IL-23, stimulates of this pathway [65].

The following will describe the effect of each of these pathways on malignancy progression.

### Wnt/β-catenin signaling’s impact on T CD4+ and T CD8+ function

T CD4+ and CD8+ lymphocytes tolerance is also increased by β-catenin in DC cells in the tumor microenvironment [66]. Moreover, LRP5 and LRP6 co-receptors have a significant deficiency in DC immune tolerance, diminishing tolerance by suppressing the Wnt pathway’s interaction with the LRP5/6 and Frizzled cortex [67]. According to research, β-catenin/mTOR/IL-10 signaling impairs DCs’ ability to cross-react with CD8+ T-cells [68]. Besides, the Wnt/β-catenin pathway influenced T CD4+ cell differentiation [69]. Thus, the induction of GATA-3 transcription factor expression by unique AT-rich sequence binding protein-1 (SATB1), a chromatin regulator that plays a vital role in T cell proliferation, TCF-1 and β-catenin proteins induces Th2 differentiation [70,71].

On the other hand, another study reported that β-catenin pathway activity in murine T CD4+ cells leads to increased RAR-related orphan receptor C (RARC) regulation and Th17 cell differentiation, thus resulting in the secretion of tumor-induced pro-inflammatory cytokines [72]. Interestingly, the TCF-1 protein increases the Th2 and Tfh cells differentiation; in contrast, it inhibits the T CD4+ differentiation into Th1 and Th17 cells [69]. It is worth noting that β-catenin will increase the differentiation and function of T-cells. As mentioned, the Wnt signaling pathway leads to the induction of immune cell tolerance in HPV infections. In this regard, β-catenin activity leads to immune escape by reducing the penetration of dendritic cells and T-cells’ dysfunction and resistance to anti-PD-1-based therapies [73].

### PD-1/PD-L1 axis’ impact on T-CD4+ and T-CD8+ function

One of the effective mechanisms in controlling and clearing viral infections is the cytotoxicity exerted by lymphocytes, especially cytotoxic T lymphocytes. Thus, activating these cells depends on two mechanisms that involve recognizing the desired antigen by the T cell receptor and the stimulator molecules [74,75]. It has been shown that PD-L1 is one of the inhibitory molecules of T lymphocytes that are expressed on APC cells and exerts its effect by binding to its PD-1 receptor on activated T-cells; as a result, it inhibits the function of T-cells [76,77]. On the other hand, the inhibitory mechanism of T-cells is triggered by PD-1 signaling in the inhibition of glycolysis and amino acid metabolism and increased fatty acid oxidation, leading to defects in these cells’ function [37,78]. Agata et al. [80] have identified that the PD-1 (known as CD279) participates in programmed cell death, in which the expression of this gene is induced quickly after signaling by the T cell receptor (TCR) and is modulated by cytokines [47,79–84].

Moreover, PD-1 also expresses on the surface of other immune cells such as B cells, natural killer cells (NK), NKT-cells, DC, and monocytes [85,86]. On this basis, after binding PD-1 to its ligand at the APCs surface, it inhibits T cell function by recruiting Src homology region 2-containing protein tyrosine phosphatase 2 (SHP2) [59,87,88], and accumulating on the T cell receptor (TCR) complex. This mechanism is due to the phosphorylation of tyrosine residues in the CD3 complex and CD28 [41]. Thus, PD-1 can inhibit the phosphorylation of the CD3z and ZAP70 chains, which is the initial stage after binding to TCR [44,89,90]. In studies, along with direct signals of TCR, CD28 activates actuation signals in T-cells by activating the PI3K pathway, and PD-1 suppresses this pathway by inhibiting the PI3K pathway [37,89]. This function is owing to the phosphorylation of immune-receptor tyrosine-based inhibitory motif (ITIM) and immune-receptor tyrosine-based switch motif (ITSM) as the PD-1 intracellular receptors [37]. The ITSM, as a crucial intracellular receptor, is phosphorylated by SHP-2, which absorbs SHP-2 tyrosine phosphatase and ultimately leads to the inactivation of PI3K and downstream inhibition of the Akt pathway [37,44,89,90]. This phosphorylation leads to dysfunction of other signaling pathways such as protein kinase B (PKB/Akt), mechanistic target of rapamycin (mTOR), RAS, MAPK/MEK, ERK, etc. It leads to decreased cytokine secretion, cell proliferation, T-cells differentiation, and eventually, their immune function attenuation [91]. Moreover, PD-1 signaling was involved in reducing of phospho-Akt regulation, mTOR, S6, ERK2, tensin homolog (PTEN), and phosphatase over-regulation [6,37]. PTEN depletion is also a common mutation in tumors that increases PI3K/Akt; subsequently, this mutation gives rise to the over-expression of PD-L1 [6].

On the other hand, T CD4+ cells infiltrate into the tumor region and exert anti-cancer activity. Also, T CD4+ cells are low exhausted than T CD8+ lymphocytes [92]. On this basis, Balança et al. found that tumor-infiltrating T CD4+ cells with high levels of PD-1 and CD39 release significant amounts of cytokines, even though these cells generate low levels of cytokines in an exhaustion situation [92]. In fact, effective cell activities such as IL-2 secretion, proliferative capacity, extracellular cytolytic activity, synthesis and secretion of TNF-α and IFN-γ have decreased in T CD8+ cells at the onset of exhaustion [93,94]. Consequently, these mechanisms, which include the overexpression of inhibitory receptors such as PD-1, contribute to the overstimulation-related destruction of these cells [95]. To put it another way, PD-1 regulates glycolytic and mitochondrial alterations, inhibiting the transcriptional coactivator PGC-1α. In actuality, PGC-1α overexpression enhances the function and metabolism of exhausted CD8 T-cells [96]. Besides, decreased mTOR activation in PD-1+ CD8 T-cells also increases the activity of the FoxO1 transcription factor, which in turn increases the expression of PD-1 and the survival of exhausted T CD8 cells [97]. On the other hand, after releasing WNT ligands by cancer cells, they cause DC cells to activate the conventional WNT signaling pathway, increasing and decreasing IL-10 and IL-12 production, respectively. In this regard, they promote T-reg cell differentiation and suppress CTLs [69]. Furthermore, GSK3 inhibition in cancer cells increases the activity of β-catenin, stabilizes PD-L1, and causes CTL exhaustion in interaction with PD-1 [69].

Moreover, tumor-specific antigens, transcription factor TOX, and CXCL13 are also expressed by T CD4+ lymphocytes carrying CD39+ and PD-1+. Blocking PD-1 has been shown to promote T CD4+ cell activity and CD154 expression, cytokine production, dendritic cell maturation, and T CD8 cell proliferation and differentiation, specific to the tumor’s surroundings [92].

## The Impact of the Wnt Signaling Pathway and PD-1/PD-L1 Axis on Tumor Infiltration Lymphocytes (TILs)

The disease state of HPV is related to the level of PD-L1 expression in such a way that in inflammatory cells infiltrating into the tumor, such as TILs, the level of PD-1/PD-L1 expression was higher in cervical cancer than in endometrial and ovarian adenocarcinoma [98]. As reported, in the PD-L1+ tumor environment, a higher number of tumor-infiltrating lymphocytes was associated with a better prognosis [98].

Tumor infiltrating lymphocyte (TILs) are one of the important components of the tumor immune microenvironment, and the quantity and function of these cells reflect the strength of the antitumor response [99–101]. Many quantitative studies have investigated the relationship between PD-L1 expression and TILs in the tumor immune microenvironment in cervical malignancy [102–104]. Feng et al. showed that in 10.0% of people with cervical cancer, the expression level of PD-L1 in TILs cells, and the secretion of inflammatory cytokines increased [105]. The evaluation of the quantity of TIL and its functional state can complement the level of PD-L1 expression in tumors since TIL can also predict the response to anti-PD-L1 therapy [106]. In cervical cancer, Karim et al. demonstrated that more than 50% of TIL expressed PD-1, and only 19% of tumor cells were PD-L1 positive [107].

Furthermore, although PD-L1 expression had no effect on patient survival directly, patients who had an overabundance of infiltrating regulatory T-cells survived longer with PD-L1-positive tumors [107]. D’Alessandris et al. reported in 2020 that stromal TIL cells have the more immunogenic potential for cervical cancer than CD3+/CD4+ helper T-cells. Also, they showed that during malignancies, 100% of immune cells and 92% of tumor cells express PD-L1 [108]. On the other hand, the number of cancer cells with PD-L1 was associated with an increase in TIL and PD-L1 expression in immune cells [108]. So, the expression of PD-L1 on the tumor cells’ surface, in addition to immune escape of cells, leads to the weakening of the anti-tumor immune response of TIL cells and, as a result, tumor progression [104]. In contrast, high PD-L1 expression in TILs is substantially associated with the existence of high TILs. It has also been demonstrated that high PD-L1 expression has a negligible association with age, tumor size, lymph node positivity, or histological grade. However, clinical trials are required to examine the impact of PD-L1 expression in the immune stroma to predict optimal treatment response [105].

Nevertheless, it was shown in the study by Goldsberry et al. [109] that there is an inverse association between the Wnt signaling pathway and TILs. This study examined the effect of neoadjuvant chemotherapy and the relationship between the Wnt signaling pathway and tumor-infiltrating lymphocytes in HGSOC [109]. Additional investigation is required to better understand the Wnt pathway’s role in immune regulation.

## The Role of Wnt Signaling and the PD-1/PD-L1 Axis in the Progression of Cancer

In the neoplasm progression trend, activated T-cells and NK cells in the tumor microenvironment (TME) released significant levels of IFN-γ [110], which leads to increased PD-L1 expression on the surface of cancer cells, subsequently enabling them to evade the immune system’s access [111,112]. Also, it has been shown that there is a strong parallel between increased PD-L1 expression and HPV infection [113]. The PD-L1 methylation pathway participates in suppressing gene expression and other cellular protein transcription in cervical cancer induced by HPV [113], and the PD-1/PD-L1 expression on tumor-infiltrating lymphocytes (TIL) in cervical cancer is more significant than in other malignancies [114].

According to the findings, numerous transcription factors, including c-MYC, STAT1, and STAT3, efficiently promote PD-L1 gene expression and the c-MYC gene is widely involved in various malignancies [7]. On the other hand, elevating the activity of the Wnt signaling pathway causes the expression of essential genes, including c-MYC (a regulator gene and proto-oncogene), Nanog (a transcription factor sustaining pluripotency of embryonic stem cells), Oct4 (octamer-binding transcription factor 4), Sox2 (sex-determining region Y-box 2), Snai1 (a zinc finger protein regulating epithelial to mesenchymal transition), and Twist (a primary helix-loop-helix transcription factor), to be increased, resulting in tumor development [115]. It has also been shown that during HPV infection, viral oncoproteins such as E5, E6, and E7 cause post-translational modifications in histones and influence the methylation state of cellular targets by influencing other cellular genes such as MYC, APOBEC3, and interacting with DNMT1 and other epigenetic modulators [64]. As a result, owing to the promotion of PD-L1 gene expression and Wnt signaling pathway activation, the progression of malignancy and tumor growth has been induced by HPV infection.

## Human Papillomaviruses

Human papillomaviruses are the double-stranded DNA viruses of the *Papillomaviridae* family. The capsid of these viruses is icosahedral, without envelope, and their genome has eight open reading frames (ORF) [116]. Papillomaviruses are epitheliotropic, dividing into mucosal and skin contaminants and classified into low-risk and high-risk varieties based on their ability to induce cancer and malignancy [117,118]. HPV-6 and HPV-11 are among the low-risk types that generate 90% of genital warts and rarely lead to malignancy [119,120]; however, HPV-16 and HPV-18 are high-risk types that cause 70% of cervical cancers [121].

Moreover, the World Health Organization (WHO) reported that cervical cancer is the fourth most common cancer among women and accounts for 30% of worldwide deaths [122]. Other malignancies associated with the virus include penis neoplasm, vulva, anus, anal, oropharyngeal, and head and neck cancer [123–126]. The papillomaviruses initiate proliferation by entering the target cells through damaged skin or mucosal squamous epithelium [127]. Thus, leading to cell division and genome replication by exploiting cellular DNA polymerase in basal cells, the infected cells are transported to the upper layers [128].

Furthermore, HPVs have two types of amplification: plasmid replication and vegetative replication [129,130]. It has also been recommended that the HPV DNA encode six early proteins (E1, E2, E4, E5, E6, and E7) and two late proteins (L1 and L2) [127] that through E5, E6, and E7 oncoproteins conduct malignancy and cellular transformation [4,65,131–133]. These oncoproteins, by accumulation in the nucleus and cytoplasm of target cells and interacting with cell pathways, lead to genomic inconsistency and result in malignancy by disrupting cell cycle controllers [134]. The stimulation of cellular signals via HPV oncoproteins after primary infection leads to the completion of viral replication and the constitution of infectious particles [1]. Also, the high expression of oncoproteins leads to the disruption of regular cell activity and stimulation of uncontrolled transformation. Interactions of E6 and E7 oncoproteins with different cellular pathways regulate signaling pathways and other mechanisms associated with HPV-caused cancers. These signaling mechanisms and pathways that are involved in persistent virus infection, such as, Hedgehog, Erk/MAPK, Notch, Wnt signaling pathways, PI3K/Akt, EGFR, and immune checkpoints, including PD-1 and CTLA-4, participate in differentiation and survival proliferation, cell cycle development [1,2,135–139]. However, the interaction of HPV oncoproteins and the PD-1/PD-L1 pathways, the regulation of the Wnt/β-catenin pathway by these oncoproteins, and their synergistic effect on disease progression, is still controversial [140].

### Wnt/β-catenin signaling in cancers associated with HPV

Several mutations in different type of cancer could occur in components of the Wnt pathway. Moreover, activating the conventional Wnt pathway is one of the essential mechanisms in epithelial cell malignancies caused by HPV infection. The HPV oncoproteins have directly and indirectly regulated these pathways [138,141]. Also, these oncoproteins target the canonical Wnt pathway; in contrast, they do not regulate the non-canonical Wnt pathway [1]. However, some components of the Wnt pathway, including Wnt7A and Dvl, the target proteins of E6 oncoprotein, are activating non-conventional Wnt pathways [1]. In HPV-induced cancers, mutations in the Catenin Beta 1 (CTNNB1) and AXIN1 genes, components of the Wnt pathway, are uncommon [142,143]. During cancer progression, membranous β-catenin decreases and accumulates in the nucleus and cytoplasm of cervical cancer and oropharyngeal squamous carcinoma cell cancer biopsies [138,144]. The progression and tumorigenesis of cervical cancer cells occur through the activation of a G protein receptor family member, called LGR5, gradually expressed in cervical cancer cells, leading to Wnt pathway activation [145]. In cervical cancer, the over-activity of the Wnt signaling system is related to the dysfunction of the GSK-3 component through Ser phosphorylation and several activities of the Wnt pathway’s components modified in the post-transcription process, contributing to the negative regulation of this pathway [146]. Moreover, cervical tumor cells have an exaggerated expression of genes that maintain and control the Wnt signaling pathway, including JUN, c-MYC, FZD2, RAC1, GSK-3β, Dvl-1, and CTNNB1 [147–149]. Also, the presence of HPV E6 and E7 oncoproteins disrupts several cellular function proteins, such as human telomerase reverse transcriptase (hTERT), p53, p300 CBP, Dvl, and PP2A, that are related to the regulation of the Wnt signaling pathway [1]. For example, in patients with oral malignancy, it has been observed that due to the high expression of β-catenin-binding FOXM1, non-recurrence is higher than in those with lower FOXM1 levels, and it is significantly promoted in the presence of the HPV genome [150]. As a result, the regulatory elements of the Wnt signaling pathway are leading to the progression of malignancy and poor prognosis in HPV-induced cancers [1]. Moreover, in oropharyngeal cancer cells, β-catenin is significantly higher in the cytoplasm and nucleus, and in contrast, in healthy cells, β-catenin is mainly found in cell junctions [151]. In this way, Sominsky et al. showed that the E6/AP complex is essential for the function of E6 in Wnt-activated cells; thus, the level of E6 is reduced through the mechanism of the proteasome, followed by the reformed reinstated of E6/AP complex [152]. The E6/AP complex induces its response independent of the catalytic function of TCF. Furthermore, the E6 protein affects the expression of myeloid zinc finger 1 (MZF1), thus activating transcription of Homeobox protein (NKX)-2, and since the FOXM1 promoter has three regions for the NKX-2 gene, it also indirectly leads to the increase of FOXM1 [153]. High levels of FOXM1 in cells expressing the E6 gene promote β-catenin transfer to the nucleus and cytoplasm. This function leads to TCF transcription activation and expression of Wnt/β-catenin target genes like c-MYC and Cyclin D1, as well as stemness-related genes like Nanog and Oct4, all of which are regulated by the MZF1/NKX2-1 transcription factor axis [153,154]. *In vitro* studies have also shown that Dvl2 cleaves the degradation complex of β-catenin and increases TCF transcriptional activity by interacting with the E6 gene [155]. On the other hand, the E7 oncogene, because of its association with its catalytic subunit, can also inhibit PP2A action by stabilizing β-catenin in the cytoplasm [1,156]. Thus, it is worth noting that identifying the mechanisms of viral oncogenes’ role in the Wnt/β-catenin signaling pathway leads to the early detection of biomarkers and targeted therapies in HPV malignancy.

### PD-1/PDL-1 axis in cancers associated with HPV

As mentioned, papillomaviruses, via their oncoproteins, stimulate cell proliferation and cell survival and modulate keratinocyte differentiation, resulting in the onset and progression of cancer [128]. Liu et al. [157] showed that in 40 cervical cancer samples, the HPV E7 oncoprotein had affected cancer cells’ escape from the immune system. They observed that the overexpression of PD-L1 leads to the inhibition of peripheral blood mononuclear cell (PBMC) products and also suppresses of T cell activity, resulting from the over-expression of oncoprotein E7 in the epithelial carcinoma of human prostate cancer cell lines (PC3) [157]. However, this study found that despite the expression of PD-L1 on the surface of cancerous cells, the non-cancerous cervical epithelium rarely expressed PD-L1 [157]. Meanwhile, in 2017 Feng et al. [158] studied 54 cervical patients infected with HPV-16 and normal cervical cytology as two experimental groups and determined the association between PD-L1 and HPV-induced cancer [158]. During this study, they found that precancerous lesions of the cervix had higher levels of PD-L1 than normal cervical cells. The excessive expression of PD-L1 leads to the persistence of HPV infection in cervical precancerous lesions [158]. Moreover, Mezache et al. found a positive association between PD-L1 expression and cervical intraepithelial neoplasia (CIN) grade [127]. On this basis, tumor cell metastasis and the CIN grade promote PD-L1 in cervical cancer [9]. Also, they observed that primary inflammatory T CD8+ lymphocytes that expressed PD-L1 were present in the vicinity of neoplastic CIN cells [127]. According to experiments conducted by Wong et al. in 2012 [8], they observed that, in 40 hr-HPV samples with various grades of CIN, in the cervical area, T-cells, DCs, INF-γ, IL-12, and IL-10 express high levels of PD-1 and PD-L1. This expression diminishes with an increasing grade of CIN via DC80 and CD86 signaling [127]. That results in plummeted levels of Th1 cytokines such as INF-γ and IL-12, also increased levels of Th2 cytokines such as IL-10 [159]. On the other hand, Qin et al. in 2017 performed research in this field and observed that HPV leads to mutations in new antigens, which play a significant role in the immune system’s inhibition. Significant changes are also demonstrated in checkpoint genes, including PD-1, PD-L1, and CTLA-4. Furthermore, PD-L1 is directly related to the enolase 1 (ENO1) regulatory genes, including positive regulatory domain I (PRDM1), OVO homologue-like 1 (OVOL1), and MNT regulators [160]. Also, in 2018, according to a study by Choschzick et al. [161], they examined 55 samples using immunohistochemistry and found no association between PD-L1 and the grade of HPV-induced cancer [161].

A study in 2020 conducted by Bucau et al. showed that in HPV-induced anal cancer, in high-grade wounds, the penetration of T CD8+ lymphocytes increases in both the lamina propria and the epithelium. In addition, it is suggested that HPV directly activates PD-1 pathways in the epithelium, inhibiting the cytotoxic and anti-tumor functions of lymphocytes [162]. Besides, PD-L1 and INF-γ mRNA levels in HPV-related HNSCC disease significantly promote tonsil plaques [127,163].

Moreover, the PD-1 ligands are selectively expressed in various cancers, tumor environments, and various malignancies such as tissue and blood cancers [164–166]. Due to the increase in PD-L1 levels during cervical HPV-associated cancer, this protein can be introduced as a biomarker [114,167].

Overall, the findings significantly suggest that HPV oncoproteins have a role in both the immune checkpoint and the canonical Wnt signaling pathway. However, some E6 target proteins, such as WNT7A and Dvl, activate the non-canonical Wnt signaling pathways, contributing to the severity of HPV-related disorders.

## Therapeutic Approaches Related to Wnt/β-Catenin Signaling and PD-1/PDL-1 Axis

Given the impact of approximately 30% of chemotherapy medicines on HPV carcinogenicity and recovery in individuals with cervical cancer, therapeutic methods such as HPV vaccination are essential to improve HPV16 and other hr-HPV infections and related malignancies [168,169]. Due to the promotion of mitosis and PD-L1 expression with increasing viral load, immunotherapy methods differ at various phases of HPV infection [61]. Several HPV therapies inhibiting the immune checkpoint have been developed to improve the vaccine’s effect on the carcinogenic process [170]. Nivolumab, pembrolizumab, and atezolizumab are monoclonal antibodies that target the PD-1/PD-L1 and CTLA-4 pathways as immunotherapy (fda.gov) [169,171]. It’s worth noting that PD-1/PD-L1 immunotherapy antibodies are related to higher numbers of tumor-infiltrating T CD8+ cells, reinvigorate anti-tumor immunity, and improve patient outcomes [172,173]. Despite this, Th17 cells and pro-inflammatory cytokine secretion like IL-17 have shown resistance to PD-1-based treatments [174–176]. In a study by Li et al. [175], in mice with lung cancer, high levels of IL-17 secretion were associated with resistance to PD-1-based therapies; additionally, it was discovered that blocking PD-1 in mice after neutralization of IL-17 as well as anti-PD-1 treatment reduced the size of cancerous tumors and increased the activity of T CD8+ cells [175]. Thus, following PD-1/PD-L1 inhibitors, glycolysis levels in cancer cells decrease, resulting in increased glucose in TME and T-cells activation [177]. Also, blocking PD-1 leads to improved CD4 TIL cell function, DC maturation, CD86 expression, and IL-12 secretion [92].

In addition to the PD-1/PD-L1 axis, the Wnt pathway is also associated with cancer metabolism and immunotherapy [115]. On this basis, it has been documented that RNA-interference (RNAi)-based therapies against the Wnt pathway increase the penetration of cytotoxic T-cells and consequently reduce tumor size [178]. It has also been observed that, by inhibiting the Wnt signaling pathway, resistance to PD-1 inhibitors and tumor cell growth is reduced [115]. Following the use of CPI, the T CD4+ and CD8+ cell lines’ functions increase due to PD-1 and PD-L1 expression and Wnt pathway activity in CD4+ and CD8+ TIL cells [115,179]. Also, the self-sufficiency of effector cells to TME, correlated with the classical Wnt active signaling pathway, contributes to cancer immunotherapy resistance [180]. In the absence of Wnt ligands, GSK3β, as a component of axin, causes the β-TrCP to degrade the PD-L1 phosphorylation-dependent proteasome and facilitate interferon-secreting CTLs to penetrate the tumor microenvironment [69]. On the other hand, the immune checkpoint inhibitor (ICI) reduces the ability of PD-1 and CTLA-4 receptors, which diminishes the penetration of CTLs and leads to a decrease in the therapeutic effect of ICI [69]. Moreover, the lack of CTLs has occurred in the active Wnt pathway tumor cells. It has been shown that the anti-CTLA4 monoclonal antibodies, in combination with canonical Wnt signaling inhibitors may prevent cancer progression in mice samples [181]. In this regard, combination therapy following the use of immune checkpoint inhibitors and anti-Wnt signaling pathway, has been shown to be effective in enhancing the therapeutic effect of malignancies [115]. Moreover, interference with GSK3 inhibitors has also been found to cause T CD8+ and Th17 cells to differentiate into anti-cancer stem cells [182,183]. Notwithstanding the effect of anti-tumor activity of antibodies against immune checkpoints, such as pembrolizumab, in HPV16+ individuals with PD-L1+ tumor cells, they had more effectiveness in combination with radiotherapy, chemotherapy or therapeutic vaccines [113]. Despite the efficacy of anti-PD-1/PD-L1 treatments, adverse effects are observed in a limited percentage of patients [184]. As a result, predictive biomarkers determine whether patients are eligible for this treatment and predict clinical outcomes [185]. For instance, in cervical cancer, inhibiting PD-1 causes T-cell exhaustion to be reversed. Despite inhibiting PD-1/PD-L1, several checkpoint molecules, including TIGIT and Tim-3, have a role in T and NK cell depletion [186,187]. Thus, the expression of PD-1 on the surface of CTLs during cervical cancer can be a crucial determinant in predicting the efficacy of PD-1 blockade therapy [188]. This therapy technique has significant adverse effects due to the immunological tolerance process and the efficiency of inhibiting immune checkpoints [184]. Immunogenicity and poor permeability of tumor tissues are both disadvantages of antibody medicines, which delay the response of PD-1/PD-L1 antibody therapies [189,190]. As previously stated, immunosuppressive therapy causes the reactivation of T-cells, resulting in tumor mortality in the mouse model; also, peptide immunization causes T-cells to reactivate and destroy tumors [184].

Generally, immunotherapy is often safer than other forms of oncology treatment, such as radiation, chemotherapy, and surgery, since it is non-invasive and relies on the ability of autoimmune cells to resist neoplasia [191]. Besides, because immune checkpoint inhibitors are particular to the target cell, they have few adverse effects [192] (Table 1).

**Table 1:** Therapeutic approach related to PDL-1 and Wnt/β-catenin signaling

Therapy	Target	Out come	References
Nivolumab Pembrolizumab Atezolizumab	PD-1 PDL-1 CTLA-4	Promotion of tumor infiltrating of T CD8+ cells Reinvigorate anti-tumor immunity Improve malignancy trend	[172,173]
Immunecheckpiont inhibitors	PD-1 CTLA-4 Tim-3 TIGIT	Reduction of glycolysis levels in cancerous cells Promotion of glucose in TME and T-cells activation Reduce ability of PD-1 and CTLA-4 receptors Depletion of T-cells and NK cells function	[177] [69] [187]
RNA-interference (RNAi)	Wnt/β-catenin	Increase penetration of cytotoxic T-cells Reduce tumor size Decrease PD-1 inhibitor resistance Tumor cell growth reduction	[178] [115]

### Wnt/β-catenin signaling pathway leads to ICI resistance therapy

In recent years, treating various solid tumors, such as lung cancer, kidney cancer, and cervical malignancy, through the inhibition of immune checkpoints PD-1/PD-L1 has achieved significant results [171,193]. However, it has been reported that the response to anti-PD-1/PD-L1 monotherapy depends on the extent of tumor-infiltrating lymphocytes [194].

Furthermore, as mentioned in previous section, the abnormal activity of the Wnt/β-catenin signaling pathway leads to increased cell proliferation, invasion, and migration and thus induces malignancy [195–197]. In this regard, regulating the activity of dendritic cells (DC), B cells and T-cells make immunotherapy effective [198]. For example, specific inhibition of the canonical Wnt signaling pathway LRP5/6 in DCs has effectively increased the effect of immunotherapy in mouse studies [199]. It has been shown that upregulation of Wnt pathway activity increases resistance to immunotherapy through the initiation of the non-T cell-inflamed tumor microenvironment (TME). Besides, increasing the activity of β-catenin pathway, in turn, inhibits the production of CCL4 and subsequently reduces the recruitment of BATF3 DCs in the TME and, as a result, reduces the activity of T-cells [200].

In other words, the Wnt/β-catenin signaling pathway, following the creation of a TME, causes a decrease in immune cells in the tumor microenvironment and subsequently reduces the therapeutic effect of ICIs [201]. In this regard, Spranger et al. [202], showed in a mouse study that there is an inverse relationship between the activity of the catenin signaling pathway and the infiltration of CD8+ T-cells. In other words, in mice with a high percentage of CD8+ T-cells, the catenin signaling pathway had little activity [202]. Furthermore, the number of CD103+ dendritic cells (DC) in tumor areas is directly related to the number of T-cells infiltrated, and IFN-cytokine secretion decreases significantly [201,203]. As a result, this mechanism reduces of antibody presentation by DCs and, subsequently, resistance to ICI treatment [201].

On the other hand, the Wnt/β-catenin signaling pathway leads to the modulation of TAMs in the TME and, subsequently, to the development of a protumoral phenotype and resistance to ICI [204,205]. In this regard, Kaler et al., in a study conducted on HCT116 and Hke-3 cancer cell lines, showed that TAMs present in the tumor area increase the activity of the Wnt/β-catenin signaling pathway, thereby inhibiting cancer cells’ apoptosis which is caused by TRAIL [205]. In other words, this increase in Wnt/β-catenin signaling pathway activity leads to an increase in the Snail gene and the induction of a tumor mesenchymal transition phenotype, which can in turn can be a reason for ICI resistance [204–206].

In addition, it has been shown that resistance to ICI can be caused by TME modulation through the interaction of TAMs with Wnt/β-catenin signaling or lactic acid production, leading to the creation of an immunosuppressive environment for cytotoxic T cells in the tumor environment [201]. In this regard, many efforts have been made to inhibit the Wnt signaling pathway and find effective therapeutic targets [207–210]. For example, in the study conducted by Ganesh et al. [211], it was observed that by inhibiting β-catenin by a β-catenin inhibitor (DCR-BCAT) that selectively knocks down the gene that transcribes β-catenin (CTNNB1) in tumor cells, it leads to a significant decrease in the activity of the Wnt/β-catenin signaling pathway, which subsequently induces T-cells and strengthens the response of checkpoint immune inhibitors [201,211].

In a phase I study, another type of WNT/β-catenin pathway inhibitor leading to disruption of PORCN (an enzyme that facilitates WNT secretion) was investigated [201]. The results indicate that following the inhibition of this WNT/β-catenin pathway along with the administration of the PD-1 monoclonal antibody spartalizumab (NCT01351103), significant results were obtained in 53% of patients who were resistant to ICI [212].

In addition to the mentioned investigators, it has been shown that natural compounds can also be used as a combination therapy in addition to effective prevention and treatment by affecting the Wnt/β-catenin signaling pathway, leading to an increase in the effect of ICI [213]. In this regard, in phase II clinical trial in patients with cervical cancer and resistant to treatment, it was observed that following the combination therapy of pembrolizumab as an anti-PD-L1 drug along with vitamin D (as a concomitant drug) and curcumin (drug complement), the anti-tumor immune response is strengthened (trial number: NCT03192059) [171,213].

## Conclusion

The development of the tumor microenvironment enhances dendritic cell signaling and type 1 interferon release. In addition, chemokines cause T CD8+ cells to infiltrate and exert authority in the TEM, followed by the production of PD-L1, IDO, Treg cell infiltration, and anergy, all of which suppress T CD8+ cells [214], and correlate to a decrease in immunosuppressive pathways [215]. Also, the native immune phenotype is inhibited by the reduced expression of inflammatory T-cell genes in tumor cells compared to normal tissue [216]. A recent study published in the cancer genome atlas (TCGA) database found a negative correlation between the expression of inflammatory genes in T-cells and tumor cells, with one of the potential mechanisms being stimulation of the Wnt/β-catenin pathway [216]. Moreover, invasive features such as lymph node metastasis or angiogenesis are associated with the up-expression of PD-1, PD-L1, and CD8; their identification could be a diagnostic marker in cervical cancer tissues [217]. As mentioned, the PD-1/PD-L1 pathway leads to the dysfunction of cytotoxic T CD8+ cells, which is a barrier to cancer treatment, and this pathway leads to the escape of tumor cells from the immune system [157]. Ultimately, given the role of the mechanisms and pathways of Wnt/β-catenin and PD-1/PD-L1 in carcinogenesis and tumor formation, both of these pathways, by affecting the c-MYC gene and consequently deactivating the functions of the T CD8+ cell, lead to tumor cells escaping from the immune system and ultimately resistant viral infection. This information can also provide a new strategy for diagnosis and the development of effective treatments.

## Abbreviation List

APCs: Antigens presenting cells
APC: Adenomatous polyposis coli
SATB1: AT-rich sequence binding protein-1
β-TrCP: Beta-transducing repeat-containing protein
Brg-1: *Brahma-related gene 1*
CD: Cluster of differentiation
c-MYC: Cellular myelocytomatosis
CIN: Cervical intraepithelial neoplasia
CREPT: Cell cycle-related and expression elevated protein in tumor
CBP/p300: CREB-binding protein
CK1: Casein kinase 1
CTNNB1: Catenin Beta 1
DC: Dendritic cells
Dlv: Disheveled
ERK: Extracellular signal-regulated kinase
EGFR: Epidermal growth factor receptor
ENO1: Enolase 1
FHL2: Four-and-a-half-LIM-domain 2
GM-CSF: Granulocyte-macrophage colony-stimulating factor
GSK-3β: Glycogen synthase kinase-3β
hTERT: Human telomerase reverse transcriptase
NKX-2: Homeobox protein-2
ICI: Immune checkpoint inhibitor
INF-γ: Interferon-gamma
IRF-1: Interferon regulatory factor-1
ITIM: Immune-receptor tyrosine-based inhibitory motif
ITSM: Immune-receptor tyrosine-based switch motif
JAK2: Janus kinase 2
LEF: Lymphoid enhancing factor
MMP-7: Metalloproteinase 7
MQ: Macrophages
MAPK: Mitogen-activated protein kinase
mTOR: Mechanistic target of rapamycin
MZF1: Myeloid zinc finger 1
NK cells: Natural killer cells
ORF: Open reading frames
OVOL1: OVO homologue-like 1
PC3: Prostate cancer cell lines
PBMC: Peripheral blood mononuclear cell
PRDM1: Positive regulatory domain I
PD-1: Programmed cell death protein 1
PTEN: Phosphatase and tensin homolog
PCP: Planer cell polarization
PP2A: Protein phosphatase 2A
PKB/Akt: Protein kinase B
RNAi: RNA-Interference
RARC: RAR-Related orphan receptor C
SHP2: Src homology region 2-containing protein tyrosine phosphatase 2
SOX6: SRY-box transcription factor 6
Ser: Serine
TIL: Tumor-infiltrating lymphocytes
TCF: T-cell factor
TAZ: Transcriptional co-activator with PDZ-binding motif
Thr: Threonine
TME: Tumor microenvironment
TCGA: The cancer genome atlas
WHO: World Health Organization
YAP: Yes-associated protein
VEGF: Vascular endothelial growth factor

## References

[1] Bello, J., Nieva, L., Paredes, A., Gonzalez, A., Zavaleta, L. et al. (2015). Regulation of the Wnt/β-catenin signaling pathway by human papillomavirus E6 and E7 oncoproteins. Viruses*,* 7 *(*8*),* 4734–4755. DOI 10.3390/v7082842. 26295406 PMC4576203

[2] Hemmat, N., Mokhtarzadeh, A., Aghazadeh, M., Jadidi-Niaragh, F., Baradaran, B. et al. (2020). Role of microRNAs in epidermal growth factor receptor signaling pathway in cervical cancer. Molecular Biology Reports*,* 47 *(*6*),* 4553–4568. DOI 10.1007/s11033-020-05494-4. 32383136

[3] Aghbash, P. S., Eslami, N., Shamekh, A., Entezari-Maleki, T., Baghi, H. B. (2021). SARS-CoV-2 infection: The role of PD-1/PD-L1 and CTLA-4 axis. Life Sciences*,* 270 *(*8*),* 119124. DOI 10.1016/j.lfs.2021.119124. 33508291 PMC7838580

[4] Wang, B., Tian, T., Kalland, K. H., Ke, X., Qu, Y. (2018). Targeting Wnt/β-catenin signaling for cancer immunotherapy. Trends in Pharmacological Sciences*,* 39 *(*7*),* 648–658. DOI 10.1016/j.tips.2018.03.008. 29678298

[5] Butte, M. J., Keir, M. E., Phamduy, T. B., Sharpe, A. H., Freeman, G. J. (2007). Programmed death-1 ligand 1 interacts specifically with the B7-1 costimulatory molecule to inhibit T cell responses. Immunity*,* 27 *(*1*),* 111–122. DOI 10.1016/j.immuni.2007.05.016. 17629517 PMC2707944

[6] Francisco, L. M., Salinas, V. H., Brown, K. E., Vanguri, V. K., Freeman, G. J. et al. (2009). PD-L1 regulates the development, maintenance, and function of induced regulatory T cells. Journal of Experimental Medicine*,* 206 *(*13*),* 3015–3029. DOI 10.1084/jem.20090847. 20008522 PMC2806460

[7] Casey, S. C., Tong, L., Li, Y., Do, R., Walz, S. et al. (2016). MYC regulates the antitumor immune response through CD47 and PD-L1. Science*,* 352 *(*6282*),* 227–231. DOI 10.1126/science.aac9935. 26966191 PMC4940030

[8] Wong, C., Chen, C., Wu, Q., Liu, Y., Zheng, P. (2015). A critical role for the regulated Wnt-myc pathway in naive T cell survival. The Journal of Immunology*,* 194 *(*1*),* 158–167. DOI 10.4049/jimmunol.1401238. 25429066 PMC4272883

[9] Yang, W., Lu, Y. P., Yang, Y. Z., Kang, J. R., Jin, Y. D. et al. (2017). Expressions of programmed death (PD)-1 and PD-1 ligand (PD-L1) in cervical intraepithelial neoplasia and cervical squamous cell carcinomas are of prognostic value and associated with human papillomavirus status. Journal of Obstetrics and Gynaecology Research*,* 43 *(*10*),* 1602–1612. DOI 10.1111/jog.13411. 28833798

[10] Spranger, S., Gajewski, T. F., Kline, J. (2016). MYC—A thorn in the side of cancer immunity. Cell Research*,* 26 *(*6*),* 639–640. DOI 10.1038/cr.2016.50. 27113275 PMC4897180

[11] Peng, X., Yang, L., Chang, H., Dai, G., Wang, F. et al. (2014). Wnt/β-catenin signaling regulates the proliferation and differentiation of mesenchymal progenitor cells through the p53 pathway. PLoS One*,* 9 *(*5*),* e97283. DOI 10.1371/journal.pone.0097283. 24819053 PMC4018322

[12] Louis, I., Heinonen, K. M., Chagraoui, J., Vainio, S., Sauvageau, G. et al. (2008). The signaling protein Wnt4 enhances thymopoiesis and expands multipotent hematopoietic progenitors through β-catenin-independent signaling. Immunity*,* 29 *(*1*),* 57–67. DOI 10.1016/j.immuni.2008.04.023. 18617424

[13] Liu, A., Chen, S., Cai, S., Dong, L., Liu, L. et al. (2014). Wnt5a through noncanonical Wnt/JNK or Wnt/PKC signaling contributes to the differentiation of mesenchymal stem cells into type II alveolar epithelial cells in vitro. PLoS One*,* 9 *(*3*),* e90229. DOI 10.1371/journal.pone.0090229. 24658098 PMC3962348

[14] Bilir, B., Kucuk, O., Moreno, C. S. (2013). Wnt signaling blockage inhibits cell proliferation and migration, and induces apoptosis in triple-negative breast cancer cells. Journal of Translational Medicine*,* 11 *(*1*),* 1–12. DOI 10.1186/1479-5876-11-280. 24188694 PMC4228255

[15] Gradl, D., Kühl, M., Wedlich, D. (1999). The Wnt/Wg signal transducer β-catenin controls fibronectin expression. Molecular and Cellular Biology*,* 19 *(*8*),* 5576–5587. DOI 10.1128/MCB.19.8.5576. 10409747 PMC84410

[16] Dollar, G. L., Weber, U., Mlodzik, M., Sokol, S. Y. (2005). Regulation of Lethal giant larvae by Dishevelled. Nature*,* 437 *(*7063*),* 1376–1380. DOI 10.1038/nature04116. 16251968

[17] Ayala-Calvillo, E., Mojica-Vázquez, L. H., García-Carrancá, A., González-Maya, L. (2018). Wnt/β-catenin pathway activation and silencing of the APC gene in HPV-positive human cervical cancer-derived cells. Molecular Medicine Reports*,* 17 *(*1*),* 200–208. DOI 10.3892/mmr.2017.7853. 29115417 PMC5780127

[18] Davidson, G., Wu, W., Shen, J., Bilic, J., Fenger, U. et al. (2005). Casein kinase 1 γ couples Wnt receptor activation to cytoplasmic signal transduction. Nature*,* 438 *(*7069*),* 867–872. DOI 10.1038/nature04170. 16341016

[19] Mao, J., Wang, J., Liu, B., Pan, W., Farr, G. H. III et al. (2001). Low-density lipoprotein receptor-related protein-5 binds to Axin and regulates the canonical Wnt signaling pathway. Molecular Cell*,* 7 *(*4*),* 801–809. DOI 10.1016/S1097-2765(01)00224-6. 11336703

[20] Shimizu, H., Julius, M. A., Giarre, M., Zheng, Z., Brown, A. et al. (1997). Transformation by Wnt family proteins correlates with regulation of beta-catenin. Cell Growth & Differentiation: The Molecular Biology Journal of the American Association for Cancer Research*,* 8 *(*12*),* 1349–1358. 9419423

[21] Hsu, S. C., Galceran, J., Grosschedl, R. (1998). Modulation of transcriptional regulation by LEF-1 in response to Wnt-1 signaling and association with β-catenin. Molecular and Cellular Biology*,* 18 *(*8*),* 4807–4818. DOI 10.1128/MCB.18.8.4807. 9671490 PMC109066

[22] Tauriello, D. V., Jordens, I., Kirchner, K., Slootstra, J. W., Kruitwagen, T. et al. (2012). Wnt/β-catenin signaling requires interaction of the Dishevelled DEP domain and C terminus with a discontinuous motif in Frizzled. PNAS*,* 109 *(*14*),* E812–E820. DOI 10.1073/pnas.1114802109. 22411803 PMC3325702

[23] Korinek, V., Barker, N., Willert, K., Molenaar, M., Roose, J. et al. (1998). Two members of the Tcf family implicated in Wnt/β-catenin signaling during embryogenesis in the mouse. Molecular and Cellular Biology*,* 18 *(*3*),* 1248–1256. DOI 10.1128/MCB.18.3.1248. 9488439 PMC108837

[24] Daniels, D. L., Weis, W. I. (2005). β-catenin directly displaces Groucho/TLE repressors from Tcf/Lef in Wnt-mediated transcription activation. Nature Structural & Molecular Biology*,* 12 *(*4*),* 364–371. DOI 10.1038/nsmb912. 15768032

[25] Zhang, Y., Liu, C., Duan, X., Ren, F., Li, S. et al. (2014). CREPT/RPRD1B, a recently identified novel protein highly expressed in tumors, enhances the β-catenin· TCF4 transcriptional activity in response to Wnt signaling. Journal of Biological Chemistry*,* 289 *(*33*),* 22589–22599. DOI 10.1074/jbc.M114.560979. 24982424 PMC4132767

[26] Labalette, C., Renard, C. A., Neuveut, C., Buendia, M. A., Wei, Y. (2004). Interaction and functional cooperation between the LIM protein FHL2, CBP/p300, and β-catenin. Molecular and Cellular Biology*,* 24*,* 10689–10702. DOI 10.1128/MCB.24.24.10689-10702.2004. 15572674 PMC533999

[27] Takemaru, K. I., Moon, R. T. (2000). The transcriptional coactivator CBP interacts with β-catenin to activate gene expression. The Journal of Cell Biology*,* 149 *(*2*),* 249–254. DOI 10.1083/jcb.149.2.249. 10769018 PMC2175158

[28] Barker, N., Hurlstone, A., Musisi, H., Miles, A., Bienz, M. et al. (2001). The chromatin remodelling factor Brg-1 interacts with β-catenin to promote target gene activation. The EMBO Journal*,* 20 *(*17*),* 4935–4943. DOI 10.1093/emboj/20.17.4935. 11532957 PMC125268

[29] Bandapalli, O. R., Dihlmann, S., Helwa, R., Macher-Goeppinger, S., Weitz, J. et al. (2009). Transcriptional activation of the β-catenin gene at the invasion front of colorectal liver metastases. The Journal of Pathology*,* 218 *(*3*),* 370–379. DOI 10.1002/path.2539. 19347947

[30] Ikeda, S., Kishida, S., Yamamoto, H., Murai, H., Koyama, S. et al. (1998). Axin, a negative regulator of the Wnt signaling pathway, forms a complex with GSK-3β and β-catenin and promotes GSK-3β-dependent phosphorylation of beta-catenin. The EMBO Journal*,* 17 *(*5*),* 1371–1384. DOI 10.1093/emboj/17.5.1371. 9482734 PMC1170485

[31] Sobrado, P., Jedlicki, A., Bustos, V. H., Allende, C. C., Allende, J. E. (2005). Basic region of residues 228-231 of protein kinase CK1α is involved in its interaction with axin: Binding to axin does not affect the kinase activity. Journal of Cellular Biochemistry*,* 94 *(*2*),* 217–224. DOI 10.1002/(ISSN)1097-4644. 15565646

[32] Papkoff, J., Rubinfeld, B., Schryver, B., Polakis, P. (1996). Wnt-1 regulates free pools of catenins and stabilizes APC-catenin complexes. Molecular and Cellular Biology*,* 16 *(*5*),* 2128–2134. DOI 10.1128/MCB.16.5.2128. 8628279 PMC231200

[33] Azzolin, L., Panciera, T., Soligo, S., Enzo, E., Bicciato, S. et al. (2014). YAP/TAZ incorporation in the β-catenin destruction complex orchestrates the Wnt response. Cell*,* 158 *(*1*),* 157–170. DOI 10.1016/j.cell.2014.06.013. 24976009

[34] Su, Y., Fu, C., Ishikawa, S., Stella, A., Kojima, M. et al. (2008). APC is essential for targeting phosphorylated β-catenin to the SCFβ-TrCP ubiquitin ligase. Molecular Cell*,* 32 *(*5*),* 652–661. DOI 10.1016/j.molcel.2008.10.023. 19061640

[35] Polakis, P. (2012). Wnt signaling in cancer. Cold Spring Harbor Perspectives in Biology*,* 4 *(*5*),* a008052. DOI 10.1101/cshperspect.a008052. PMC3331705 22438566

[36] Laba, S., Mallett, G., Amarnath, S. (2021). The depths of PD-1 function within the tumor microenvironment beyond CD8+ T-cells. Seminars in Cancer Biology*,* 86*,* 1045–1055. DOI 10.1016/j.semcancer.2021.05.022 Elsevier. 34048897

[37] Chinai, J. M., Janakiram, M., Chen, F., Chen, W., Kaplan, M. et al. (2015). New immunotherapies targeting the PD-1 pathway. Trends in Pharmacological Sciences*,* 36 *(*9*),* 587–595. DOI 10.1016/j.tips.2015.06.005. 26162965 PMC4562806

[38] Chemnitz, J. M., Parry, R. V., Nichols, K. E., June, C. H., Riley, J. L. (2004). SHP-1 and SHP-2 associate with immunoreceptor tyrosine-based switch motif of programmed death 1 upon primary human T cell stimulation, but only receptor ligation prevents T cell activation. The Journal of Immunology*,* 173 *(*2*),* 945–954. DOI 10.4049/jimmunol.173.2.945. 15240681

[39] Lipson, E. J., Vincent, J. G., Loyo, M., Kagohara, L. T., Luber, B. S. et al. (2013). PD-L1 expression in the Merkel cell carcinoma microenvironment: Association with inflammation, Merkel cell polyomavirus, and overall survival. Cancer Immunology Research*,* 1 *(*1*),* 54–63. DOI 10.1158/2326-6066.CIR-13-0034. 24416729 PMC3885978

[40] Freeman, G. J., Long, A. J., Iwai, Y., Bourque, K., Chernova, T. et al. (2000). Engagement of the PD-1 immunoinhibitory receptor by a novel B7 family member leads to negative regulation of lymphocyte activation. The Journal of Experimental Medicine*,* 192 *(*7*),* 1027–1034. DOI 10.1084/jem.192.7.1027. 11015443 PMC2193311

[41] Schönrich, G., Raftery, M. J. (2019). The PD-1/PD-L1 axis and virus infections: a delicate balance. Frontiers in Cellular and Infection Microbiology*,* 9*,* 207. DOI 10.3389/fcimb.2019.00207. 31263684 PMC6584848

[42] Nguyen, L. T., Ohashi, P. S. (2015). Clinical blockade of PD1 and LAG3—potential mechanisms of action. Nature Reviews Immunology*,* 15 *(*1*),* 45–56. DOI 10.1038/nri3790. 25534622

[43] Strome, S. E., Dong, H., Tamura, H., Voss, S. G., Flies, D. B. et al. (2003). B7-H1 blockade augments adoptive T-cell immunotherapy for squamous cell carcinoma. Cancer Research*,* 63 *(*19*),* 6501–6505. 14559843

[44] Yokosuka, T., Takamatsu, M., Kobayashi-Imanishi, W., Hashimoto-Tane, A., Azuma, M. et al. (2012). Programmed cell death 1 forms negative costimulatory microclusters that directly inhibit T cell receptor signaling by recruiting phosphatase SHP2. Journal of Experimental Medicine*,* 209 *(*6*),* 1201–1217. DOI 10.1084/jem.20112741. 22641383 PMC3371732

[45] Konishi, J., Yamazaki, K., Azuma, M., Kinoshita, I., Dosaka-Akita, H. et al. (2004). B7-H1 expression on non-small cell lung cancer cells and its relationship with tumor-infiltrating lymphocytes and their PD-1 expression. Clinical Cancer Research*,* 10 *(*15*),* 5094–5100. DOI 10.1158/1078-0432.CCR-04-0428. 15297412

[46] Rozali, E. N., Hato, S. V., Robinson, B. W., Lake, R. A., Lesterhuis, W. J. (2012). Programmed death ligand 2 in cancer-induced immune suppression. Clinical and Developmental Immunology*,* 2012 *(*2*),* 1–8. DOI 10.1155/2012/656340. PMC3350956 22611421

[47] Yamazaki, T., Akiba, H., Iwai, H., Matsuda, H., Aoki, M. et al. (2002). Expression of programmed death 1 ligands by murine T-cells and APC. The Journal of Immunology*,* 169 *(*10*),* 5538–5545. DOI 10.4049/jimmunol.169.10.5538. 12421930

[48] Latchman, Y., Wood, C. R., Chernova, T., Chaudhary, D., Borde, M. et al. (2001). PD-L2 is a second ligand for PD-1 and inhibits T cell activation. Nature Immunology*,* 2 *(*3*),* 261–268. DOI 10.1038/85330. 11224527

[49] Lee, S. J., Jang, B. C., Lee, S. W., Yang, Y. I., Suh, S. I. et al. (2006). Interferon regulatory factor-1 is prerequisite to the constitutive expression and IFN-gamma-induced upregulation of B7-H1 (CD274). FEBS Letters*,* 580 *(*3*),* 755–762. DOI 10.1016/j.febslet.2005.12.093. 16413538

[50] Kataoka, K., Shiraishi, Y., Takeda, Y., Sakata, S., Matsumoto, M. et al. (2016). Aberrant PD-L1 expression through 3′-UTR disruption in multiple cancers. Nature*,* 534 *(*7607*),* 402–406. DOI 10.1038/nature18294. 27281199

[51] George, J., Saito, M., Tsuta, K., Iwakawa, R., Shiraishi, K. et al. (2017). Genomic amplification of CD274 (PD-L1) in small-cell lung cancer. Clinical Cancer Research*,* 23 *(*5*),* 1220–1226. DOI 10.1158/1078-0432.CCR-16-1069. 27620277 PMC6329376

[52] Dong, P., Xiong, Y., Yu, J., Chen, L., Tao, T. et al. (2018). Control of PD-L1 expression by miR-140/142/340/383 and oncogenic activation of the OCT4-miR-18a pathway in cervical cancer. Oncogene*,* 37 *(*39*),* 5257–5268. DOI 10.1038/s41388-018-0347-4. 29855617 PMC6160397

[53] Akbay, E. A., Koyama, S., Carretero, J., Altabef, A., Tchaicha, J. H. et al. (2013). Activation of the PD-1 pathway contributes to immune escape in EGFR-driven lung tumors. Cancer Discovery*,* 3 *(*12*),* 1355–1363. DOI 10.1158/2159-8290.CD-13-0310. 24078774 PMC3864135

[54] Concha-Benavente, F., Srivastava, R. M., Trivedi, S., Lei, Y., Chandran, U. et al. (2016). Identification of the cell-intrinsic and-extrinsic pathways downstream of EGFR and IFNγ that induce PD-L1 expression in head and neck cancer. Cancer Research*,* 76 *(*5*),* 1031–1043. DOI 10.1158/0008-5472.CAN-15-2001. 26676749 PMC4775348

[55] Parsa, A. T., Waldron, J. S., Panner, A., Crane, C. A., Parney, I. F. et al. (2007). Loss of tumor suppressor PTEN function increases B7-H1 expression and immunoresistance in glioma. Nature Medicine*,* 13 *(*1*),* 84–88. DOI 10.1038/nm1517. 17159987

[56] Baruah, P., Bullenkamp, J., Wilson, P. O., Lee, M., Kaski, J. C. et al. (2019). TLR9 mediated tumour-stroma interactions in human papilloma virus (HPV)-positive head and neck squamous cell carcinoma up-regulate PD-L1 and PD-L2. Frontiers in Immunology*,* 10*,* 1644. DOI 10.3389/fimmu.2019.01644. 31379843 PMC6648892

[57] Nishimura, H., Nose, M., Hiai, H., Minato, N., Honjo, T. (1999). Development of lupus-like autoimmune diseases by disruption of the PD-1 gene encoding an ITIM motif-carrying immunoreceptor. Immunity*,* 11 *(*2*),* 141–151. DOI 10.1016/S1074-7613(00)80089-8. 10485649

[58] Fife, B. T., Pauken, K. E. (2011). The role of the PD-1 pathway in autoimmunity and peripheral tolerance. Annals of the New York Academy of Sciences*,* 1217 *(*1*),* 45–59. DOI 10.1111/j.1749-6632.2010.05919.x. 21276005

[59] Okazaki, T., Chikuma, S., Iwai, Y., Fagarasan, S., Honjo, T. (2013). A rheostat for immune responses: The unique properties of PD-1 and their advantages for clinical application. Nature Immunology*,* 14 *(*12*),* 1212–1218. DOI 10.1038/ni.2762. 24240160

[60] Honda, T., Egen, J. G., Lämmermann, T., Kastenmüller, W., Torabi-Parizi, P. et al. (2014). Tuning of antigen sensitivity by T cell receptor-dependent negative feedback controls T cell effector function in inflamed tissues. Immunity*,* 40 *(*2*),* 235–247. DOI 10.1016/j.immuni.2013.11.017. 24440150 PMC4792276

[61] Usta, C. S., Altun, E., Afsar, S., Bulbul, C. B., Usta, A. et al. (2020). Overexpression of programmed cell death ligand 1 in patients with CIN and its correlation with human papillomavirus infection and CIN persistence. Infectious Agents and Cancer*,* 15 *(*1*),* 1–10. DOI 10.1186/s13027-020-00312-9. 32695218 PMC7367318

[62] Zhao, D. M., Yu, S., Zhou, X., Haring, J. S., Held, W. et al. (2010). Constitutive activation of Wnt signaling favors generation of memory CD8 T-cells. The Journal of Immunology*,* 184 *(*3*),* 1191–1199. DOI 10.4049/jimmunol.0901199. 20026746 PMC2809813

[63] Gaffen, S. L. (2009). Structure and signalling in the IL-17 receptor family. Nature Reviews Immunology*,* 9 *(*8*),* 556–567. DOI 10.1038/nri2586. PMC2821718 19575028

[64] Mecocci, S., Porcellato, I., Armando, F., Mechelli, L., Brachelente, C. et al. (2021). Equine genital squamous cell carcinoma associated with EcPV2 infection: RANKL pathway correlated to inflammation and Wnt signaling activation. Biology*,* 10 *(*3*),* 244. DOI 10.3390/biology10030244. 33801021 PMC8003831

[65] Aghbash, P. S., Hemmat, N., Nahand, J. S., Shamekh, A., Memar, M. Y. et al. (2021). The role of Th17 cells in viral infections. International Immunopharmacology*,* 91 *(*5*),* 107331. DOI 10.1016/j.intimp.2020.107331. 33418239

[66] Ruan, Y., Ogana, H., Gang, E., Kim, H. N., Kim, Y. M. (2021). Wnt signaling in the tumor microenvironment, in tumor microenvironment*,* pp. 107–121. Springer. 10.1007/978-3-030-47189-7_7 PMC8477804 33123996

[67] Hong, Y., Manoharan, I., Suryawanshi, A., Shanmugam, A., Swafford, D. et al. (2016). Deletion of LRP5 and LRP6 in dendritic cells enhances antitumor immunity. Oncoimmunology*,* 5 *(*4*),* e1115941. DOI 10.1080/2162402X.2015.1115941. 27141399 PMC4839371

[68] Fu, C., Liang, X., Cui, W., Ober-Blöbaum, J. L., Vazzana, J. et al. (2015). β-Catenin in dendritic cells exerts opposite functions in cross-priming and maintenance of CD8+ T-cells through regulation of IL-10. PNAS*,* 112 *(*9*),* 2823–2828. DOI 10.1073/pnas.1414167112. 25730849 PMC4352820

[69] Li, X., Xiang, Y., Li, F., Yin, C., Li, B. et al. (2019). WNT/β-catenin signaling pathway regulating T cell-inflammation in the tumor microenvironment. Frontiers in Immunology*,* 10*,* 2293. DOI 10.3389/fimmu.2019.02293. 31616443 PMC6775198

[70] Notani, D., Gottimukkala, K. P., Jayani, R. S., Limaye, A. S., Damle, M. V. et al. (2010). Global regulator SATB1 recruits β-catenin and regulates TH2 differentiation in Wnt-dependent manner. PLoS Biology*,* 8 *(*1*),* e1000296. DOI 10.1371/journal.pbio.1000296. 20126258 PMC2811152

[71] Yu, Q., Sharma, A., Oh, S. Y., Moon, H. -G., Hossain, M. Z. et al. (2009). T cell factor 1 initiates the T helper type 2 fate by inducing the transcription factor GATA-3 and repressing interferon-γ. Nature Immunology*,* 10 *(*9*),* 992–999. DOI 10.1038/ni.1762. 19648923 PMC2824257

[72] Keerthivasan, S., Aghajani, K., Dose, M., Molinero, L., Khan, M. W. et al. (2014). Wnt/β-catenin signaling in T-cells drives epigenetic imprinting of pro-inflammatory properties and promotes colitis and colon cancer. Science Translational Medicine*,* 6 *(*225*),* 225ra28. DOI 10.1126/scitranslmed.3007607. PMC4020714 24574339

[73] de Galarreta, M. R., Bresnahan, E., Molina-Sánchez, P., Lindblad, K. E., Maier, B. et al. (2019). β-catenin activation promotes immune escape and resistance to anti-PD-1 therapy in hepatocellular carcinoma. Cancer Discovery*,* 9 *(*8*),* 1124–1141. DOI 10.1158/2159-8290.CD-19-0074. 31186238 PMC6677618

[74] Stanley, M. (2009). Immune responses to human papilloma viruses. Indian Journal of Medical Research*,* 130 *(*3*),* 266–276. 19901436

[75] Flies, D. B., Sandler, B. J., Sznol, M., Chen, L. (2011). Blockade of the B7-H1/PD-1 pathway for cancer immunotherapy. The Yale Journal of Biology and Medicine*,* 84 *(*4*),* 409–421. 22180678 PMC3238327

[76] Selenko-Gebauer, N., Majdic, O., Szekeres, A., Höfler, G., Guthann, E. et al. (2003). B7-H1 (programmed death-1 ligand) on dendritic cells is involved in the induction and maintenance of T cell anergy. The Journal of Immunology*,* 170 *(*7*),* 3637–3644. DOI 10.4049/jimmunol.170.7.3637. 12646628

[77] Dong, H., Strome, S. E., Salomao, D. R., Tamura, H., Hirano, F. et al. (2002). Tumor-associated B7-H1 promotes T-cell apoptosis: A potential mechanism of immune evasion. Nature Medicine*,* 8 *(*8*),* 793–800. DOI 10.1038/nm730. 12091876

[78] Patsoukis, N., Bardhan, K., Chatterjee, P., Sari, D., Liu, B. et al. (2015). PD-1 alters T-cell metabolic reprogramming by inhibiting glycolysis and promoting lipolysis and fatty acid oxidation. Nature Communications*,* 6 *(*1*),* 1–13. DOI 10.1038/ncomms7692. PMC4389235 25809635

[79] Ishida, Y., Agata, Y., Shibahara, K., Honjo, T. (1992). Induced expression of PD-1, a novel member of the immunoglobulin gene superfamily, upon programmed cell death. The EMBO Journal*,* 11 *(*11*),* 3887–3895. DOI 10.1002/j.1460-2075.1992.tb05481.x. 1396582 PMC556898

[80] Agata, Y., Kawasaki, A., Nishimura, H., Ishida, Y., Tsubat, T. et al. (1996). Expression of the PD-1 antigen on the surface of stimulated mouse T and B lymphocytes. International Immunology*,* 8 *(*5*),* 765–772. DOI 10.1093/intimm/8.5.765. 8671665

[81] Wherry, E. J., Ha, S. J., Kaech, S. M., Haining, W. N., Sarkar, S. et al. (2007). Molecular signature of CD8+ T cell exhaustion during chronic viral infection. Immunity*,* 27 *(*4*),* 670–684. DOI 10.1016/j.immuni.2007.09.006. 17950003

[82] Chikuma, S., Terawaki, S., Hayashi, T., Nabeshima, R., Yoshida, T. et al. (2009). PD-1-mediated suppression of IL-2 production induces CD8+ T cell anergy *in vivo*. The Journal of Immunology*,* 182 *(*11*),* 6682–6689. DOI 10.4049/jimmunol.0900080. 19454662

[83] Ahn, E., Araki, K., Hashimoto, M., Li, W., Riley, J. L. et al. (2018). Role of PD-1 during effector CD8 T cell differentiation. PNAS*,* 115 *(*18*),* 4749–4754. DOI 10.1073/pnas.1718217115. 29654146 PMC5939075

[84] Terawaki, S., Chikuma, S., Shibayama, S., Hayashi, T., Yoshida, T. et al. (2011). IFN-α directly promotes programmed cell death-1 transcription and limits the duration of T cell-mediated immunity. The Journal of Immunology*,* 186 *(*5*),* 2772–2779. DOI 10.4049/jimmunol.1003208. 21263073

[85] Sharpe, A. H., Wherry, E. J., Ahmed, R., Freeman, G. J. (2007). The function of programmed cell death 1 and its ligands in regulating autoimmunity and infection. Nature Immunology*,* 8 *(*3*),* 239–245. DOI 10.1038/ni1443. 17304234

[86] Keir, M. E., Butte, M. J., Freeman, G. J., Sharpe, A. H. (2008). PD-1 and its ligands in tolerance and immunity. Annual Review of Immunology*,* 26 *(*4*),* 677–704. DOI 10.1146/annurev.immunol.26.021607.090331. PMC10637733 18173375

[87] Carracedo, A., Cantley, L. C., Pandolfi, P. P. (2013). Cancer metabolism: Fatty acid oxidation in the limelight. Nature Reviews Cancer*,* 13 *(*4*),* 227–232. DOI 10.1038/nrc3483. 23446547 PMC3766957

[88] Sharpe, A. H., Pauken, K. E. (2018). The diverse functions of the PD1 inhibitory pathway. Nature Reviews Immunology*,* 18 *(*3*),* 153–167. DOI 10.1038/nri.2017.108. 28990585

[89] Parry, R. V., Chemnitz, J. M., Frauwirth, K. A., Lanfranco, A. R., Braunstein, I. et al. (2005). CTLA-4 and PD-1 receptors inhibit T-cell activation by distinct mechanisms. Molecular and Cellular Biology*,* 25 *(*21*),* 9543–9553. DOI 10.1128/MCB.25.21.9543-9553.2005. 16227604 PMC1265804

[90] Sheppard, K. A., Fitz, L. J., Lee, J. M., Benander, C., George, J. A. et al. (2004). PD-1 inhibits T-cell receptor induced phosphorylation of the ZAP70/CD3ζ signalosome and downstream signaling to PKCθ. FEBS Letters*,* 574 *(*1–3*),* 37–41. DOI 10.1016/j.febslet.2004.07.083. 15358536

[91] Hui, E., Cheung, J., Zhu, J., Su, X., Taylor, M. J. et al. (2017). T cell costimulatory receptor CD28 is a primary target for PD-1-mediated inhibition. Science*,* 355 *(*6332*),* 1428–1433. DOI 10.1126/science.aaf1292. 28280247 PMC6286077

[92] Balança, C. C., Salvioni, A., Scarlata, C. M., Michelas, M., Martinez-Gomez, C. et al. (2021). PD-1 blockade restores helper activity of tumor-infiltrating, exhausted PD-1hiCD39+ CD4 T-cells. JCI Insight*,* 6 *(*2*),* 3604. DOI 10.1172/jci.insight.142513. PMC7934837 33332284

[93] Wherry E. J., Blattman J. N., Murali-Krishna K., van der Most R., Ahmed R. (2003). Viral persistence alters CD8 T-cell immunodominance and tissue distribution and results in distinct stages of functional impairment. Journal of Virology*,* 77 *(*8*),* 4911–4927. DOI 10.1128/JVI.77.8.4911-4927.2003. 12663797 PMC152117

[94] Pauken, K. E., Wherry, E. J. (2015). Overcoming T cell exhaustion in infection and cancer. Trends in Immunology*,* 36 *(*4*),* 265–276. DOI 10.1016/j.it.2015.02.008. 25797516 PMC4393798

[95] Hashimoto, M., Kamphorst, A. O., Im, S. J., Kissick, H. T., Pillai, R. N. et al. (2018). CD8 T cell exhaustion in chronic infection and cancer: Opportunities for interventions. Annual Review of Medicine*,* 69 *(*1*),* 301–318. DOI 10.1146/annurev-med-012017-043208. 29414259

[96] Bengsch, B., Johnson, A. L., Kurachi, M., Odorizzi, P. M., Pauken, K. E. et al. (2016). Bioenergetic insufficiencies due to metabolic alterations regulated by the inhibitory receptor PD-1 are an early driver of CD8+ T cell exhaustion. Immunity*,* 45 *(*2*),* 358–373. DOI 10.1016/j.immuni.2016.07.008. 27496729 PMC4988919

[97] Staron, M. M., Gray, S. M., Marshall, H. D., Parish, I. A., Chen, J. H. et al. (2014). The transcription factor FoxO1 sustains expression of the inhibitory receptor PD-1 and survival of antiviral CD8+ T-cells during chronic infection. Immunity*,* 41 *(*5*),* 802–814. DOI 10.1016/j.immuni.2014.10.013. 25464856 PMC4270830

[98] Saglam, O., Conejo-Garcia, J. (2018). PD-1/PD-L1 immune checkpoint inhibitors in advanced cervical cancer. Integrative Cancer Science and Therapeutics*,* 5 *(*2*)*. DOI 10.15761/ICST.1000272. PMC6016855 29955379

[99] Mellati, M., Eaton, K. D., Brooks-Worrell, B. M., Hagopian, W. A., Martins, R. et al. (2015). Anti-PD-1 and anti-PDL-1 monoclonal antibodies causing type 1 diabetes. Diabetes Care*,* 38 *(*9*),* e137–e138. DOI 10.2337/dc15-0889. 26116720

[100] Li, X. F., Jiang, X. Q., Zhang, J. W., Jia, Y. J. (2016). Association of the programmed cell death-1 PD1. 5 C> T polymorphism with cervical cancer risk in a Chinese population. Genetics and Molecular Research*,* 15 *(*1*),* 10–4238. DOI 10.1158/1078-0432.CCR-09-1652. 27050970

[101] Kamata, T., Suzuki, A., Mise, N., Ihara, F., Takami, M. et al. (2016). Blockade of programmed death-1/programmed death ligand pathway enhances the antitumor immunity of human invariant natural killer T-cells. Cancer Immunology, Immunotherapy*,* 65 *(*12*),* 1477–1489. DOI 10.1007/s00262-016-1901-y. 27631416 PMC5099366

[102] Cao, J., Brouwer, N. J., Richards, K. E., Marinkovic, M., van Duinen, S. et al. (2017). PD-L1/PD-1 expression and tumor-infiltrating lymphocytes in conjunctival melanoma. Oncotarget*,* 8 *(*33*),* 54722–54734. DOI 10.18632/oncotarget.18039. 28903377 PMC5589616

[103] Resch, I., Shariat, S. F., Gust, K. M. (2018). PD-1 and PD-L1 inhibitors after platinum-based chemotherapy or in first-line therapy in cisplatin-ineligible patients. Memo-Magazine of European Medical Oncology*,* 11 *(*1*),* 43–46. DOI 10.1007/s12254-018-0396-y. PMC5862914 29606979

[104] Chen, S. C., Chang, P. M. H., Wang, H. J., Tai, S. K., Chu, P. Y. et al. (2018). PD‐L1 expression is associated with p16INK4A expression in non‐oropharyngeal head and neck squamous cell carcinoma. Oncology Letters*,* 15 *(*2*),* 2259–2265. DOI 10.3892/ol.2017.7564. 29434933 PMC5776927

[105] Feng, M., Xu, L., He, Y., Sun, L., Zhang, Y. et al. (2018). Clinical significance of PD-L1 (CD274) enhanced expression in cervical squamous cell carcinoma. International Journal of Clinical and Experimental Pathology*,* 11 *(*11*),* 5370–5378. 31949618 PMC6963033

[106] Festino, L., Botti, G., Lorigan, P., Masucci, G. V., Hipp, J. D. et al. (2016). Cancer treatment with anti-PD-1/PD-L1 agents: Is PD-L1 expression a biomarker for patient selection? Drugs*,* 76 *(*9*),* 925–945. DOI 10.1007/s40265-016-0588-x. 27229745

[107] Karim, R., Jordanova, E. S., Piersma, S. J., Kenter, G. G., Chen, L. et al. (2009). Tumor-expressed B7-H1 and B7-DC in relation to PD-1+ T-cell infiltration and survival of patients with cervical carcinoma. Clinical Cancer Research*,* 15 *(*20*),* 6341–6347. DOI 10.1158/1078-0432.CCR-09-1652. 19825956

[108] D’Alessandris, N., Palaia, I., Pernazza, A., Tomao, F., Di Pinto, A. et al. (2021). PD-L1 expression is associated with tumor infiltrating lymphocytes that predict response to NACT in squamous cell cervical cancer. Virchows Archiv*,* 478 *(*3*),* 517–525. DOI 10.1007/s00428-020-02922-5. 32915266

[109] Goldsberry, W., Phillips, R. A., Montgomery, A. M., Katre, A. A., Doo, D. W. et al. (2019). Correlation between Wnt signaling and T-cell infiltration in high-grade serous ovarian cancer. Gynecologic Oncology*,* 154*,* 72. DOI 10.1016/j.ygyno.2019.04.170.31000471

[110] Sun, C., Mezzadra, R., Schumacher, T. N. (2018). Regulation and function of the PD-L1 checkpoint. Immunity*,* 48 *(*3*),* 434–452. DOI 10.1016/j.immuni.2018.03.014. 29562194 PMC7116507

[111] Yi, M., Niu, M., Xu, L., Luo, S., Wu, K. (2021). Regulation of PD-L1 expression in the tumor microenvironment. Journal of Hematology & Oncology*,* 14 *(*1*),* 1–13. DOI 10.1186/s13045-020-01027-5. 33413496 PMC7792099

[112] Li, X., Shao, C., Shi, Y., Han, W. (2018). Lessons learned from the blockade of immune checkpoints in cancer immunotherapy. Journal of Hematology & Oncology*,* 11 *(*1*),* 1–26. DOI 10.1186/s13045-018-0578-4. 29482595 PMC6389077

[113] Verhoeven, Y., Quatannens, D., Trinh, X. B., Wouters, A., Smits, E. L. et al. (2021). Targeting the PD-1 axis with pembrolizumab for recurrent or metastatic cancer of the uterine cervix: A brief update. International Journal of Molecular Sciences*,* 22 *(*4*),* 1807. DOI 10.3390/ijms22041807. 33670397 PMC7917788

[114] Mezache, L., Paniccia, B., Nyinawabera, A., Nuovo, G. J. (2015). Enhanced expression of PD L1 in cervical intraepithelial neoplasia and cervical cancers. Modern Pathology*,* 28 *(*12*),* 1594–1602. DOI 10.1038/modpathol.2015.108. 26403783

[115] El-Sahli, S., Xie, Y., Wang, L., Liu, S. (2019). Wnt signaling in cancer metabolism and immunity. Cancers*,* 11 *(*7*),* 904. DOI 10.3390/cancers11070904. 31261718 PMC6678221

[116] Zur Hausen, H. (2002). Papillomaviruses and cancer: From basic studies to clinical application. Nature Reviews Cancer*,* 2 *(*5*),* 342–350. DOI 10.1038/nrc798. 12044010

[117] Bzhalava, D., Guan, P., Franceschi, S., Dillner, J., Clifford, G. (2013). A systematic review of the prevalence of mucosal and cutaneous human papillomavirus types. Virology*,* 445 *(*1–2*),* 224–231. DOI 10.1016/j.virol.2013.07.015. 23928291

[118] Mistry, N., Wibom, C., Evander, M. (2008). Cutaneous and mucosal human papillomaviruses differ in net surface charge, potential impact on tropism. Virology Journal*,* 5 *(*1*),* 118. DOI 10.1186/1743-422X-5-118. 18854037 PMC2571092

[119] de Oliveira, G. R., Siqueira, J. D., Finger-Jardim, F., Vieira, V. C., Silva, R. L. et al. (2017). Characterisation of complete high-and low-risk human papillomavirus genomes isolated from cervical specimens in southern Brazil. Memórias do Instituto Oswaldo Cruz*,* 112 *(*10*),* 728–731. DOI 10.1590/0074-02760170121. 28954002 PMC5607523

[120] Muñoz, N., Bosch, F. X., de Sanjosé, S., Herrero, R., Castellsagué, X. et al. (2003). Epidemiologic classification of human papillomavirus types associated with cervical cancer. New England Journal of Medicine*,* 348 *(*6*),* 518–527. DOI 10.1056/NEJMoa021641. 12571259

[121] Smith, J. S., Lindsay, L., Hoots, B., Keys, J., Franceschi, S. et al. (2007). Human papillomavirus type distribution in invasive cervical cancer and high-grade cervical lesions: A meta-analysis update. International Journal of Cancer*,* 121 *(*3*),* 621–632. DOI 10.1002/(ISSN)1097-0215. 17405118

[122] WHO. (2018). Cervical cancer. https://www.who.int/cancer/prevention/diagnosis-screening/cervical-cancer/en/.

[123] Moody, C. A., Laimins, L. A. (2010). Human papillomavirus oncoproteins: Pathways to transformation. Nature Reviews Cancer*,* 10 *(*8*),* 550–560. DOI 10.1038/nrc2886. 20592731

[124] Niya, M. H. K., Tameshkel, F. S., Panahi, M., Salim, F. B., Monavari, S. H. R. et al. (2017). Human papillomavirus investigation in head and neck squamous cell carcinoma: Initial report from the low risk HPV types associations. Asian Pacific Journal of Cancer Prevention*,* 18 *(*9*),* 2573–2579. DOI 10.22034/APJCP.2017.18.9.2573. 28952562 PMC5720669

[125] D’Souza, G., Kreimer, A. R., Viscidi, R., Pawlita, M., Fakhry, C. et al. (2007). Case–control study of human papillomavirus and oropharyngeal cancer. New England Journal of Medicine*,* 356 *(*19*),* 1944–1956. DOI 10.1056/NEJMoa065497. 17494927

[126] Daling, J. R., Madeleine, M. M., Johnson, L. G., Schwartz, S. M., Shera, K. A. et al. (2004). Human papillomavirus, smoking, and sexual practices in the etiology of anal cancer. Cancer*,* 101 *(*2*),* 270–280. DOI 10.1002/(ISSN)1097-0142. 15241823

[127] Mofrad, M. G., Maleki, D. T., Faghihloo, E. (2020). The roles of programmed death ligand 1 in virus-associated cancers. Infection, Genetics and Evolution*,* 84 *(*1*),* 104368. DOI 10.1016/j.meegid.2020.104368. 32470632

[128] Pol, S. B. V., Klingelhutz, A. J. (2013). Papillomavirus E6 oncoproteins. Virology*,* 445 *(*1–2*),* 115–137. DOI 10.1016/j.virol.2013.04.026. 23711382 PMC3783570

[129] Conway, M., Meyers, C. (2009). Replication and assembly of human papillomaviruses. Journal of Dental Research*,* 88 *(*4*),* 307–317. DOI 10.1177/0022034509333446. 19407149 PMC3317948

[130] Ozbun, M. A., Meyers, C. (1997). Characterization of late gene transcripts expressed during vegetative replication of human papillomavirus type 31b. Journal of Virology*,* 71 *(*7*),* 5161–5172. DOI 10.1128/jvi.71.7.5161-5172.1997. 9188583 PMC191751

[131] Riley, R. R., Duensing, S., Brake, T., Münger, K., Lambert, P. F. et al. (2003). Dissection of human papillomavirus E6 and E7 function in transgenic mouse models of cervical carcinogenesis. Cancer Research*,* 63 *(*16*),* 4862–4871. 12941807

[132] Song, S., Pitot, H. C., Lambert, P. F. (1999). The human papillomavirus type 16 E6 gene alone is sufficient to induce carcinomas in transgenic animals. Journal of Virology*,* 73 *(*7*),* 5887–5893. DOI 10.1128/JVI.73.7.5887-5893.1999. 10364340 PMC112649

[133] Hemmat, N., Baghi, H. B. (2018). Human papillomavirus E5 protein, the undercover culprit of tumorigenesis. Infectious Agents and Cancer*,* 13 *(*1*),* 31. DOI 10.1186/s13027-018-0208-3. 30455726 PMC6230221

[134] Sashiyama, H., Shino, Y., Kawamata, Y., Tomita, Y., Ogawa, N. et al. (2001). Immortalization of human esophageal keratinocytes by E6 and E7 of human papillomavirus type 16. International Journal of Oncology*,* 19 *(*1*),* 97–103. DOI 10.3892/ijo.19.1.97. 11408928

[135] Samarzija, I., Beard, P. (2012). Hedgehog pathway regulators influence cervical cancer cell proliferation, survival and migration. Biochemical and Biophysical Research Communications*,* 425 *(*1*),* 64–69. DOI 10.1016/j.bbrc.2012.07.051. 22820185

[136] Branca, M., Ciotti, M., Santini, D., Di Bonito, L., Benedetto, A. et al. (2004). Activation of the ERK/MAP kinase pathway in cervical intraepithelial neoplasia is related to grade of the lesion but not to high-risk human papillomavirus, virus clearance, or prognosis in cervical cancer. American Journal of Clinical Pathology*,* 122 *(*6*),* 902–911. DOI 10.1309/VQXFT880JXC7QD2W. 15539382

[137] Weijzen, S., Zlobin, A., Braid, M., Miele, L., Kast, W. M. (2003). HPV16 E6 and E7 oncoproteins regulate Notch-1 expression and cooperate to induce transformation. Journal of Cellular Physiology*,* 194 *(*3*),* 356–362. DOI 10.1002/(ISSN)1097-4652. 12548555

[138] Uren, A., Fallen, S., Yuan, H., Usubütün, A., Küçükali, T. et al. (2005). Activation of the canonical Wnt pathway during genital keratinocyte transformation: A model for cervical cancer progression. Cancer Research*,* 65 *(*14*),* 6199–6206. DOI 10.1158/0008-5472.CAN-05-0455. 16024621

[139] Manzo-Merino, J., Contreras-Paredes, A., Vázquez-Ulloa, E., Rocha-Zavaleta, L., Fuentes-Gonzalez, A. M. et al. (2014). The role of signaling pathways in cervical cancer and molecular therapeutic targets. Archives of Medical Research*,* 45 *(*7*),* 525–539. DOI 10.1016/j.arcmed.2014.10.008. 25450584

[140] Allouch, S., Malki, A., Allouch, A., Gupta, I., Vranic, S. et al. (2020). High-risk HPV oncoproteins and PD-1/PD-L1 interplay in human cervical cancer: Recent evidence and future directions. Frontiers in Oncology*,* 10*,* 1228. DOI 10.3389/fonc.2020.00914. 32695664 PMC7338567

[141] Bulut, Gl, Fallen, S., Beauchamp, E. M., Drebing, L. E., Sun, J. et al. (2011). Beta-catenin accelerates human papilloma virus type-16 mediated cervical carcinogenesis in transgenic mice. PLoS One*,* 6 *(*11*),* e27243. DOI 10.1371/journal.pone.0027243. 22087269 PMC3210148

[142] Webster, M. T., Rozycka, M., Sara, E., Davis, E., Smalley, M. et al. (2000). Sequence variants of the axin gene in breast, colon, and other cancers: An analysis of mutations that interfere with GSK3 binding. Genes, Chromosomes and Cancer*,* 28 *(*4*),* 443–453. DOI 10.1002/(ISSN)1098-2264. 10862053

[143] Su, T. H., Chang, J. G., Yeh, K. T., Lin, T. H., Lee, T. P. et al. (2003). Mutation analysis of CTNNB1 (β-catenin) and AXIN1, the components of Wnt pathway, in cervical carcinomas. Oncology Reports*,* 10 *(*5*),* 1195–1200. DOI 10.3892/or.10.5.1195. 12883680

[144] Rodríguez-Sastre, M. A., González-Maya, L., Delgado, R., Lizano, M., Tsubaki, G. et al. (2005). Abnormal distribution of E-cadherin and β-catenin in different histologic types of cancer of the uterine cervix. Gynecologic Oncology*,* 97 *(*2*),* 330–336. DOI 10.1016/j.ygyno.2004.12.062. 15863126

[145] Chen, Q., Cao, H. Z., Zheng, P. S. (2014). LGR5 promotes the proliferation and tumor formation of cervical cancer cells through the Wnt/β-catenin signaling pathway. Oncotarget*,* 5 *(*19*),* 9092–9105. DOI 10.18632/oncotarget.2377. 25193857 PMC4253421

[146] Rath, G., Jawanjal, P., Salhan, S., Nalliah, M., Dhawan, I. (2015). Clinical significance of inactivated glycogen synthase kinase 3β in HPV-associated cervical cancer: Relationship with Wnt/β-C atenin pathway activation. American Journal of Reproductive Immunology*,* 73 *(*5*),* 460–478. DOI 10.1111/aji.12346. 25532422

[147] van der Meide, W. F., Snellenberg, S., Meijer, C. J., Baalbergen, A., Helmerhorst, T. J. et al. (2011). Promoter methylation analysis of WNT/β-catenin signaling pathway regulators to detect adenocarcinoma or its precursor lesion of the cervix. Gynecologic Oncology*,* 123 *(*1*),* 116–122. DOI 10.1016/j.ygyno.2011.06.015. 21726894

[148] Pérez-Plasencia, C., Vázquez-Ortiz, G., López-Romero, R., Piña-Sanchez, P., Moreno, J. et al. (2007). Genome wide expression analysis in HPV16 cervical cancer: Identification of altered metabolic pathways. Infectious Agents and Cancer*,* 2 *(*1*),* 1–10. DOI 10.1186/1750-9378-2-16. 17822553 PMC2034543

[149] Fragoso-Ontiveros, V., María Alvarez-García, R., Contreras-Paredes, A., Vaca-Paniagua, F., Alonso Herrera, L. et al. (2012). Gene expression profiles induced by E6 from non-European HPV18 variants reveals a differential activation on cellular processes driving to carcinogenesis. Virology*,* 432 *(*1*),* 81–90. DOI 10.1016/j.virol.2012.05.029. 22743128

[150] Chen, P. M., Cheng, Y. W., Wang, Y. C., Wu, T. C., Chen, C. Y. et al. (2014). Up-regulation of FOXM1 by E6 oncoprotein through the MZF1/NKX2-1 axis is required for human papillomavirus–associated tumorigenesis. Neoplasia*,* 16 *(*11*),* 961–971. DOI 10.1016/j.neo.2014.09.010. 25425970 PMC4240922

[151] Rampias, T., Boutati, E., Pectasides, E., Sasaki, C., Kountourakis, P. et al. (2010). Activation of Wnt signaling pathway by human papillomavirus E6 and E7 oncogenes in HPV16-positive oropharyngeal squamous carcinoma cells. Molecular Cancer Research*,* 8 *(*3*),* 433–443. DOI 10.1158/1541-7786.MCR-09-0345. 20215420

[152] Sominsky, S., Kuslansky, Y., Shapiro, B., Jackman, A., Haupt, Y. et al. (2014). HPV16 E6 and E6AP differentially cooperate to stimulate or augment Wnt signaling. Virology*,* 468 *(*12*),* 510–523. DOI 10.1016/j.virol.2014.09.007. 25262469

[153] Wang, B., Li, X., Liu, L., Wang, M. (2020). β-Catenin: Oncogenic role and therapeutic target in cervical cancer. Biological Research*,* 53 *(*1*),* 1–11. DOI 10.1186/s40659-020-00301-7. 32758292 PMC7405349

[154] Chen, P. M., Cheng, Y. W., Wang, Y. C., Wu, T. C., Chen, C. Y. et al. (2014). Up-regulation of FOXM1 by E6 oncoprotein through the MZF1/NKX2-1 axis is required for human papillomavirus-associated tumorigenesis. Neoplasia*,* 16 *(*11*),* 961–971. DOI 10.1016/j.neo.2014.09.010. 25425970 PMC4240922

[155] Bonilla-Delgado, J., Bulut, G., Liu, X., Cortés-Malagón, E. M., Schlegel, R. et al. (2012). The E6 oncoprotein from HPV16 enhances the canonical Wnt/β-catenin pathway in skin epidermis *in vivo*. Molecular Cancer Research*,* 10 *(*2*),* 250–258. DOI 10.1158/1541-7786.MCR-11-0287. 22160870 PMC3332097

[156] Pim, D., Massimi, P., Dilworth, S. M., Banks, L. (2005). Activation of the protein kinase B pathway by the HPV-16 E7 oncoprotein occurs through a mechanism involving interaction with PP2A. Oncogene*,* 24 *(*53*),* 7830–7838. DOI 10.1038/sj.onc.1208935. 16044149

[157] Liu, C., Lu, J., Tian, H., Du, W., Zhao, L. et al. (2017). Increased expression of PD-L1 by the human papillomavirus 16 E7 oncoprotein inhibits anticancer immunity. Molecular Medicine Reports*,* 15 *(*3*),* 1063–1070. DOI 10.3892/mmr.2017.6102. 28075442 PMC5367331

[158] Feng, Y. C., Cheng, Z. Z., Huang, Y. C., Ma, X. M. (2017). Association between PD-L1 and HPV status and the prognostic value for HPV treatment in premalignant cervical lesion patients. Medicine*,* 96 *(*25*),* e7270. DOI 10.1097/MD.0000000000007270. 28640134 PMC5484242

[159] Yang, W., Song, Y., Lu, Y. L., Sun, J. Z., Wang, H. W. (2013). Increased expression of programmed death (PD)-1 and its ligand PD-L1 correlates with impaired cell-mediated immunity in high-risk human papillomavirus-related cervical intraepithelial neoplasia. Immunology*,* 139 *(*4*),* 513–522. DOI 10.1111/imm.12101. 23521696 PMC3719068

[160] Qin, Y., Ekmekcioglu, S., Forget, M. A., Szekvolgyi, L., Hwu, P. et al. (2017). Cervical cancer neoantigen landscape and immune activity is associated with human papillomavirus master regulators. Frontiers in Immunology*,* 8*,* 689. DOI 10.3389/fimmu.2017.00689. 28670312 PMC5473350

[161] Choschzick, M., Gut, A., Fink, D. (2018). PD-L1 receptor expression in vulvar carcinomas is HPV-independent. Virchows Archiv*,* 473 *(*4*),* 513–516. DOI 10.1007/s00428-018-2364-7. 29736798

[162] Bucau, M., Gault, N., Sritharan, N., Valette, E., Charpentier, C. et al. (2020). PD-1/PD-L1 expression in anal squamous intraepithelial lesions. Oncotarget*,* 11 *(*39*),* 3582–3589. DOI 10.18632/oncotarget.27756. 33062194 PMC7533075

[163] Franzen, A., Vogt, T. J., Müller, T., Dietrich, J., Schröck, A. et al. (2018). PD-L1 (CD274) and PD-L2 (PDCD1LG2) promoter methylation is associated with HPV infection and transcriptional repression in head and neck squamous cell carcinomas. Oncotarget*,* 9 *(*1*),* 641–650. DOI 10.18632/oncotarget.23080. 29416641 PMC5787495

[164] Vranic, S., Ghosh, N., Kimbrough, J., Bilalovic, N., Bender, R. et al. (2016). PD-L1 status in refractory lymphomas. PLoS One*,* 11 *(*11*),* e0166266. DOI 10.1371/journal.pone.0166266. 27861596 PMC5115714

[165] Xu-Monette, Z. Y., Zhou, J., Young, K. H. (2018). PD-1 expression and clinical PD-1 blockade in B-cell lymphomas. Blood, The Journal of the American Society of Hematology*,* 131 *(*1*),* 68–83. DOI 10.1182/blood-2017-07-740993. PMC5755041 29118007

[166] Noh, B. J., Kwak, J. Y., Eom, D. W. (2020). Immune classification for the PD-L1 expression and tumour-infiltrating lymphocytes in colorectal adenocarcinoma. BMC Cancer*,* 20 *(*1*),* 58. DOI 10.1186/s12885-020-6553-9. 31992245 PMC6986059

[167] Kooshkaki, O., Derakhshani, A., Safarpour, H., Najafi, S., Vahedi, P. et al. (2020). The latest findings of PD-1/PD-L1 inhibitor application in gynecologic cancers. International Journal of Molecular Sciences*,* 21 *(*14*),* 5034. DOI 10.3390/ijms21145034. 32708748 PMC7404077

[168] Markman, M. (2013). Chemoradiation in the management of cervix cancer: Current status and future directions. Oncology*,* 84 *(*4*),* 246–250. DOI 10.1159/000346804. 23392268

[169] Peng, S., Tan, M., Li, Y. D., Cheng, M. A., Farmer, E. et al. (2021). PD-1 blockade synergizes with intratumoral vaccination of a therapeutic HPV protein vaccine and elicits regression of tumor in a preclinical model. Cancer Immunology, Immunotherapy*,* 70 *(*4*),* 1049–1062. DOI 10.1007/s00262-020-02754-x. 33108473 PMC7979473

[170] Haddon, C., Lewis, J. (1996). Early ear development in the embryo of the Zebrafish, *Danio rerio*. Journal of Comparative Neurology*,* 365 *(*1*),* 113–128. DOI 10.1002/(ISSN)1096-9861. 8821445

[171] Aghbash, P., Hemmat, N., Fathi, H., Baghi, H. (2022). Monoclonal antibodies in cervical malignancy-related HPV. Frontiers in Oncology*,* 12*,* 1. DOI 10.3389/fonc.2022.904790. PMC9582116 36276117

[172] Forde, P. M., Chaft, J. E., Smith, K. N., Anagnostou, V., Cottrell, T. R. et al. (2018). Neoadjuvant PD-1 blockade in resectable lung cancer. New England Journal of Medicine*,* 378 *(*21*),* 1976–1986. DOI 10.1056/NEJMoa1716078. 29658848 PMC6223617

[173] Topalian, S. L., Hodi, F. S., Brahmer, J. R., Gettinger, S. N., Smith, D. C. et al. (2012). Safety, activity, and immune correlates of anti-PD-1 antibody in cancer. New England Journal of Medicine*,* 366 *(*26*),* 2443–2454. DOI 10.1056/NEJMoa1200690. 22658127 PMC3544539

[174] Akbay, E. A., Koyama, S., Liu, Y., Dries, R., Bufe, L. E. et al. (2017). Interleukin-17A promotes lung tumor progression through neutrophil attraction to tumor sites and mediating resistance to PD-1 blockade. Journal of Thoracic Oncology*,* 12 *(*8*),* 1268–1279. DOI 10.1016/j.jtho.2017.04.017. 28483607 PMC5532066

[175] Li, Q., Ngo, P. T., Egilmez, N. K. (2021). Anti-PD-1 antibody-mediated activation of type 17 T-cells undermines checkpoint blockade therapy. Cancer Immunology, Immunotherapy*,* 70 *(*6*),* 1789–1796. DOI 10.1007/s00262-020-02795-2. 33245376 PMC10991855

[176] Ritzmann, F., Jungnickel, C., Vella, G., Kamyschnikow, A., Herr, C. et al. (2019). IL-17C-mediated innate inflammation decreases the response to PD-1 blockade in a model of Kras-driven lung cancer. Scientific Reports*,* 9 *(*1*),* 1–11. DOI 10.1038/s41598-019-46759-8. 31316109 PMC6637115

[177] Chang, C. H., Qiu, J., O’Sullivan, D., Buck, M. D., Noguchi, T. et al. (2015). Metabolic competition in the tumor microenvironment is a driver of cancer progression. Cell*,* 162 *(*6*),* 1229–1241. DOI 10.1016/j.cell.2015.08.016. 26321679 PMC4864363

[178] Kerdidani, D., Chouvardas, P., Arjo, A. R., Giopanou, I., Ntaliarda, G. et al. (2019). Wnt1 silences chemokine genes in dendritic cells and induces adaptive immune resistance in lung adenocarcinoma. Nature Communications*,* 10 *(*1*),* 1–16. DOI 10.1038/s41467-019-09370-z. PMC6441097 30926812

[179] Wen, Y. A., Xiong, X., Scott, T., Li, A. T., Wang, C. et al. (2019). The mitochondrial retrograde signaling regulates Wnt signaling to promote tumorigenesis in colon cancer. Cell Death & Differentiation*,* 26 *(*10*),* 1955–1969. DOI 10.1038/s41418-018-0265-6. 30659235 PMC6748256

[180] Galluzzi, L., Yamazaki, T., Kroemer, G. (2018). Linking cellular stress responses to systemic homeostasis. Nature Reviews Molecular Cell Biology*,* 19 *(*11*),* 731–745. DOI 10.1038/s41580-018-0068-0. 30305710

[181] Holtzhausen, A., Zhao, F., Evans, K. S., Tsutsui, M., Orabona, C. et al. (2015). Melanoma-derived Wnt5a promotes local dendritic-cell expression of IDO and immunotolerance: Opportunities for pharmacologic enhancement of immunotherapy. Cancer Immunology Research*,* 3 *(*9*),* 1082–1095. DOI 10.1158/2326-6066.CIR-14-0167. 26041736 PMC4927300

[182] Majchrzak, K., Nelson, M. H., Bowers, J. S., Bailey, S. R., Wyatt, M. M. et al. (2017). β-catenin and PI3Kδ inhibition expands precursor Th17 cells with heightened stemness and antitumor activity. JCI Insight*,* 2 *(*8*),* 5241. DOI 10.1172/jci.insight.90547. PMC5396523 28422756

[183] Gattinoni, L., Zhong, X. S., Palmer, D. C., Ji, Y., Hinrichs, C. S. et al. (2009). Wnt signaling arrests effector T cell differentiation and generates CD8+ memory stem cells. Nature Medicine*,* 15 *(*7*),* 808–813. DOI 10.1038/nm.1982. PMC2707501 19525962

[184] De Sousa Linhares, A., Leitner, J., Grabmeier-Pfistershammer, K., Steinberger, P. (2018). Not all immune checkpoints are created equal. Frontiers in Immunology*,* 9*,* 21. DOI 10.3389/fimmu.2018.01909. 30233564 PMC6127213

[185] Darvin, P., Toor, S. M., Nair, V. S., Elkord, E. (2018). Immune checkpoint inhibitors: Recent progress and potential biomarkers. Experimental & Molecular Medicine*,* 50 *(*12*),* 1–11. DOI 10.1038/s12276-018-0191-1. PMC6292890 30546008

[186] Solorzano-Ibarra, F., Alejandre-Gonzalez, A. G., Ortiz-Lazareno, P. C., Bastidas-Ramirez, B. E., Zepeda-Moreno, A. et al. (2021). Immune checkpoint expression on peripheral cytotoxic lymphocytes in cervical cancer patients: Moving beyond the PD-1/PD-L1 axis. Clinical & Experimental Immunology*,* 204 *(*1*),* 78–95. DOI 10.1111/cei.13561. 33306195 PMC7944364

[187] Zhang, Q., Bi, J., Zheng, X., Chen, Y., Wang, H. et al. (2018). Blockade of the checkpoint receptor TIGIT prevents NK cell exhaustion and elicits potent anti-tumor immunity. Nature Immunology*,* 19 *(*7*),* 723–732. DOI 10.1038/s41590-018-0132-0. 29915296

[188] Heeren, A., Rotman, J., Stam, A., Pocorni, N., Gassama, A. et al. (2019). Efficacy of PD-1 blockade in cervical cancer is related to a CD8+ FoxP3+ CD25+ T-cell subset with operational effector functions despite high immune checkpoint levels. Journal for Immunotherapy of Cancer*,* 7 *(*1*),* 1–14. DOI 10.1186/s40425-019-0526-z. 30755279 PMC6373123

[189] Naidoo, J., Page, D., Li, B. T., Connell, L. C., Schindler, K. et al. (2015). Toxicities of the anti-PD-1 and anti-PD-L1 immune checkpoint antibodies. Annals of Oncology*,* 26 *(*12*),* 2375–2391. DOI 10.1093/annonc/mdv383. 26371282 PMC6267867

[190] Wu, Q., Jiang, L., Li, S. C, He, Q. J, Yang, B. et al. (2021). Small molecule inhibitors targeting the PD-1/PD-L1 signaling pathway. Acta Pharmacologica Sinica*,* 42 *(*1*),* 1–9. DOI 10.1038/s41401-020-0366-x. 32152439 PMC7921448

[191] Khan, M., Lin, J., Liao, G., Tian, Y., Liang, Y. et al. (2018). Comparative analysis of immune checkpoint inhibitors and chemotherapy in the treatment of advanced non-small cell lung cancer: A meta-analysis of randomized controlled trials. Medicine*,* 97 *(*33*),* e11936. DOI 10.1097/MD.0000000000011936. 30113497 PMC6113026

[192] Makuku, R., Khalili, N., Razi, S., Keshavarz-Fathi, M., Rezaei, N. (2021). Current and future perspectives of PD-1/PDL-1 blockade in cancer immunotherapy. Journal of Immunology Research*,* 2021 *(*4*),* 1–15. DOI 10.1155/2021/6661406. PMC7925068 33681388

[193] Shi, J., Li, F., Luo, M., Wei, J., Liu, X. (2017). Distinct roles of Wnt/β-catenin signaling in the pathogenesis of chronic obstructive pulmonary disease and idiopathic pulmonary fibrosis. Mediators of Inflammation*,* 2017 *(*2*),* 1–16. DOI 10.1155/2017/3520581. PMC5447271 28588349

[194] Tewari, D., Bawari, S., Sharma, S., DeLiberto, L. K., Bishayee, A. (2021). Targeting the crosstalk between canonical Wnt/β-catenin and inflammatory signaling cascades: A novel strategy for cancer prevention and therapy. Pharmacology & Therapeutics*,* 227 *(*3*),* 107876. DOI 10.1016/j.pharmthera.2021.107876. 33930452

[195] Kwon, C., Cheng, P., King, I. N., Andersen, P., Shenje, L. et al. (2011). Notch post-translationally regulates β-catenin protein in stem and progenitor cells. Nature Cell Biology*,* 13 *(*10*),* 1244–1251. DOI 10.1038/ncb2313. 21841793 PMC3187850

[196] Raisch, J., Côté-Biron, A., Langlois, M. J., Leblanc, C., Rivard, N. (2021). Unveiling the roles of low-density lipoprotein receptor-related protein 6 in intestinal homeostasis, regeneration and oncogenesis. Cells*,* 10 *(*7*),* 1792. DOI 10.3390/cells10071792. 34359960 PMC8307932

[197] Gómez-Orte, E., Sáenz-Narciso, B., Moreno, S., Cabello, J. (2013). Multiple functions of the noncanonical Wnt pathway. Trends in Genetics*,* 29 *(*9*),* 545–553. DOI 10.1016/j.tig.2013.06.003. 23846023

[198] Yamamoto, D., Oshima, H., Wang, D., Takeda, H., Kita, K. et al. (2022). Characterization of RNF43 frameshift mutations that drive Wnt ligand-and R-spondin-dependent colon cancer. The Journal of Pathology*,* 257 *(*1*),* 39–52. DOI 10.1002/path.5868. 35040131 PMC9314865

[199] Zhang, Y., Wang, X. (2020). Targeting the Wnt/β-catenin signaling pathway in cancer. Journal of Hematology & Oncology*,* 13 *(*1*),* 1–16. DOI 10.1186/s13045-020-00990-3. 33276800 PMC7716495

[200] Steinhart, Z., Angers, S. (2018). Wnt signaling in development and tissue homeostasis. Development*,* 145 *(*11*),* dev146589. DOI 10.1242/dev.146589. 29884654

[201] Chehrazi-Raffle, A., Dorff, T. B., Pal, S. K., Lyou, Y. (2021). Wnt/β-catenin signaling and immunotherapy resistance: Lessons for the treatment of urothelial carcinoma. Cancers*,* 13 *(*4*),* 889. DOI 10.3390/cancers13040889. 33672668 PMC7924395

[202] Spranger, S., Bao, R., Gajewski, T. F. (2015). Melanoma-intrinsic β-catenin signalling prevents anti-tumour immunity. Nature*,* 523 *(*7559*),* 231–235. DOI 10.1038/nature14404. 25970248

[203] Shiri Aghbash, P., Shirvaliloo, M., Kasho, A. K. A., Alinezhad, F., Nauwynck, H. et al. (2022). Cluster of differentiation frequency on antigen presenting-cells: The next step to cervical cancer prognosis? International Immunopharmacology*,* 108*,* 108896. DOI 10.1016/j.intimp.2022.108896. 35640377

[204] Ding Y., Shen S., Lino A. C., Curotto de Lafaille M. A., Lafaille J. J. (2008). Beta-catenin stabilization extends regulatory T cell survival and induces anergy in nonregulatory T-cells. Nature Medicine*,* 14 *(*2*),* 162–169. DOI 10.1038/nm1707. 18246080

[205] Kaler, P., Augenlicht, L., Klampfer, L. (2012). Activating mutations in β-catenin in colon cancer cells alter their interaction with macrophages; the role of snail. PLoS One*,* 7 *(*9*),* e45462. DOI 10.1371/journal.pone.0045462. 23029025 PMC3448637

[206] Horn, L. A., Riskin, J., Hempel, H. A., Fousek, K., Lind, H. et al. (2020). Simultaneous inhibition of CXCR1/2, TGF-β, and PD-L1 remodels the tumor and its microenvironment to drive antitumor immunity. Journal for Immunotherapy of Cancer*,* 8 *(*1*),* e000326. DOI 10.1136/jitc-2019-000326. 32188703 PMC7078948

[207] Lyou, Y., Habowski, A. N., Chen, G. T., Waterman, M. L. (2017). Inhibition of nuclear Wnt signalling: Challenges of an elusive target for cancer therapy. British Journal of Pharmacology*,* 174 *(*24*),* 4589–4599. DOI 10.1111/bph.13963. 28752891 PMC5727325

[208] Le, P. N., McDermott, J. D., Jimeno, A. (2015). Targeting the Wnt pathway in human cancers: Therapeutic targeting with a focus on OMP-54F28. Pharmacology & Therapeutics*,* 146 *(*4*),* 1–11. DOI 10.1016/j.pharmthera.2014.08.005. 25172549 PMC4304994

[209] Mita, M. M., Becerra, C., Richards, D. A., Mita, A. C., Shagisultanova, E. et al. (2016). Phase 1b study of WNT inhibitor vantictumab (VAN, human monoclonal antibody) with paclitaxel (P) in patients (pts) with 1st-to 3rd-line metastatic HER2-negative breast cancer (BC). Journal of Clinical Oncology*,* 34 *(*15_suppl*),* 2516. DOI 10.1200/JCO.2016.34.15_suppl.2516.27269942

[210] Kahn, M. (2014). Can we safely target the WNT pathway? Nature Reviews Drug Discovery*,* 13 *(*7*),* 513–532. DOI 10.1038/nrd4233. 24981364 PMC4426976

[211] Ganesh, S., Shui, X., Craig, K. P., Park, J., Wang, W. et al. (2018). RNAi-mediated β-catenin inhibition promotes T cell infiltration and antitumor activity in combination with immune checkpoint blockade. Molecular Therapy*,* 26 *(*11*),* 2567–2579. DOI 10.1016/j.ymthe.2018.09.005. 30274786 PMC6225018

[212] Janku, F., de Vos, F., de Miguel, M., Forde, P., Ribas, A. et al. (2020). Abstract CT034: Phase I study of WNT974+ spartalizumab in patients (pts) with advanced solid tumors. Cancer Research*,* 80 *(*16_Supplement*),* CT034. DOI 10.1158/1538-7445.am2020-ct034.

[213] Sferrazza, G., Corti, M., Brusotti, G., Pierimarchi, P., Temporini, C. et al. (2020). Nature-derived compounds modulating Wnt/β-catenin pathway: A preventive and therapeutic opportunity in neoplastic diseases. Acta Pharmaceutica Sinica B*,* 10 *(*10*),* 1814–1834. DOI 10.1016/j.apsb.2019.12.019. 33163337 PMC7606110

[214] Liu, Q., Zhu, H., Tiruthani, K., Shen, L., Chen, F. et al. (2018). Nanoparticle-mediated trapping of Wnt family member 5A in tumor microenvironments enhances immunotherapy for B-Raf proto-oncogene mutant melanoma. ACS Nano*,* 12 *(*2*),* 1250–1261. DOI 10.1021/acsnano.7b07384. 29370526 PMC5834397

[215] Gajewski, T. F., Schreiber, H., Fu, Y. X. (2013). Innate and adaptive immune cells in the tumor microenvironment. Nature Immunology*,* 14 *(*10*),* 1014–1022. DOI 10.1038/ni.2703. 24048123 PMC4118725

[216] Luke, J. J., Bao, R., Sweis, R. F., Spranger, S., Gajewski, T. F. (2019). WNT/β-catenin pathway activation correlates with immune exclusion across human cancers. Clinical Cancer Research*,* 25 *(*10*),* 3074–3083. DOI 10.1158/1078-0432.CCR-18-1942. 30635339 PMC6522301

[217] Wimberly, H., Brown, J. R., Schalper, K., Haack, H., Silver, M. R. et al. (2015). PD-L1 expression correlates with tumor-infiltrating lymphocytes and response to neoadjuvant chemotherapy in breast cancer. Cancer Immunology Research*,* 3 *(*4*),* 326–332. DOI 10.1158/2326-6066.CIR-14-0133. 25527356 PMC4390454

